# Diversity of microbial, biocontrol agents and nematode abundance on a susceptible *Prunus* rootstock under a *Meloidogyne* root gradient infection

**DOI:** 10.3389/fpls.2024.1386535

**Published:** 2024-09-23

**Authors:** Ilenia Clavero-Camacho, Alba N. Ruiz-Cuenca, Carolina Cantalapiedra-Navarrete, Pablo Castillo, Juan E. Palomares-Rius

**Affiliations:** ^1^ Institute for Sustainable Agriculture (IAS), Spanish National Research Council (CSIC), Cordoba, Spain; ^2^ Instituto de Estudios de Postgrado, Departamento de Agronomía, Universidad de Córdoba, Cordoba, Spain; ^3^ Departament of Animal Plant Biology and Ecology, Universidad de Jaén, Jaén, Spain

**Keywords:** metabarcoding, *Meloidogyne*, 16S rRNA, ITS, biological control

## Abstract

Root-knot nematodes (RKNs) of the genus *Meloidogyne* are one of the most damaging genera to cultivated woody plants with a worldwide distribution. The knowledge of the soil and rhizosphere microbiota of almonds infested with *Meloidogyne* could help to establish new sustainable and efficient management strategies. However, the soil microbiota interaction in deciduous woody plants infected with RKNs is scarcely studied. This research was carried out in six commercial almond groves located in southern Spain and infested with different levels of *Meloidogyne* spp. within each grove. Several parameters were measured: nematode assemblages, levels and biocontrol agents in *Meloidogyne*’s eggs, levels of specific biocontrol agents in rhizoplane and soil, levels of bacteria and fungi in rhizoplane and soil, fungal and bacterial communities by high-throughput sequencing of internal transcribed spacer (ITS), and 16S rRNA gene in soil and rhizosphere of the susceptible almond hybrid rootstock GF-677 infested with *Meloidogyne* spp. The studied almond groves showed soil degradation by nematode assemblies and fungi:bacterial ratio. Fungal parasites of *Meloidogyne* eggs were found in 56.25% of the samples. However, the percentage of parasitized eggs by fungi ranged from 1% to 8%. Three fungal species were isolated from *Meloidogyne* eggs, specifically *Pochonia chlamydosporia*, *Purpureocillium lilacinum*, and *Trichoderma asperellum*. The diversity and composition of the microbial communities were more affected by the sample type (soil vs rhizosphere) and by the geographical location of the samples than by the *Meloidogyne* density, which could be explained by the vigorous hybrid rootstock GF-677 and a possible dilution effect. However, the saprotrophic function in the functional guilds of the fungal ASV was increased in the highly infected roots vs the low infected roots. These results indicate that the presence of biocontrol agents in almond fields and the development of new management strategies could increase their populations to control partially RKN infection levels.

## 1 Introduction

Almond (*Prunus dulcis*) is a representative tree of the Mediterranean countryside and Spain. In terms of extension, the area dedicated to almond cultivation in Spain is around 744,000 ha, with more than 30% of this area located in Andalusia, Southern Spain (MAPA, 2022). Behind this crop, there is a whole industry of great economic importance that has led Spain to be the second largest producer of almonds in the world (FAOSTAT, 2021). In the last decades, almond consumption has increased worldwide due to its high nutritional and eating quality. The growing demand for almonds worldwide has promoted the expansion of intensive almond groves characterized by a high density of trees per hectare, irrigation, fertilization, new cultivars and rootstocks from breeding programs, new pruning systems, and pest and disease management (Micke, 1996). The key to success of the establishment of the new intensive almond groves was strongly supported by the selection of new rootstocks with higher tolerance to iron chlorosis and greater resistance/tolerance to pests and diseases, and self-compatible cultivars (Ortega and Dicenta, 2003; Jiménez et al., 2008; Rubio-Cabetas et al., 2017). Rootstocks play an essential role in the successful establishment and maintenance of an almond grove, as tolerance to soil-borne diseases depend upon them. The main soil-borne pathogens that can compromise tree growth and grove yield, even causing the death of young trees, include the fungi *Armillaria mellea* and *Phytophtora* spp. and plant-parasitic nematodes (PPNs) (Nyczepir, 1991; Baumgartner et al., 2011; Browne, 2017). Four nematode genera have been reported as major diseases causing severe losses in yield and quality in *Prunus* spp. grove, specifically root-knot (*Meloidogyne* spp.), root-lesion (*Pratylenchus* spp.), dagger (*Xiphinema* spp.), and ring (*Criconemoides* spp.) nematodes (Nyczepir, 1991). Rootstock breeding programs have focused on three species of *Meloidogyne* (*Meloidogyne arenaria*, *Meloidogyne incognita*, and *Meloidogyne javanica*) and one of *Pratylenchus* (*Pratylenchus vulnus*), which can become important limiting factors for this crop in warm environments (Alcañiz et al., 1996; Pinochet et al., 1996; Esmenjaud et al., 1997).

Focusing on root-knot nematodes (RKNs) (*Meloidogyne* spp.), these are obligate plant parasites with a worldwide distribution and a wide host range (Jones et al., 2013). These nematodes are sedentary endoparasites characterized by inducing the formation of galls on the roots of susceptible plants (Moens et al., 2009). The nematode parasitism causes stunted plant growth, reduced fruit quality, and lower yields. In the Mediterranean region, the most widespread RKNs are *Meloidogyne arenaria*, *M. incognita*, and *M. javanica*, although other species have also been reported affecting *Prunus* groves. Specifically, *Meloidogyne floridensis* (Handoo et al., 2004)), *Meloidogyne hispanica* (Hirschmann, 1986), and *Meloidogyne morocciensis* (Rammah and Hirschmann, 1990) were described parasitizing peach in Florida, Spain, and Morocco, respectively. Recently, *M. floridensis* was reported infecting almond groves in California (Westphal et al., 2019). As for other PPNs, control methods and management strategies against RKNs include prevention measures and methods to reduce nematode population, such as crop rotation, solarization, nematicides, resistant/tolerant rootstocks, and biological control (Verdejo-Lucas and Talavera, 2009). However, some of these methods have limitations due to the wide host range of most PPN affecting almond groves and the fact that woody crops remain for decades in the same field. Moreover, in Spain, there are no authorized nematicides for use on *Prunus* crops (MAPA, 2023). To establish a plantation, resistant rootstocks to RKNs are the most economical, sustainable, and effective method to manage the most harmful *Meloidogyne* spp (Saucet et al., 2016). In Spain, the most commonly used rootstock is the hybrid GF-677 (*Prunus dulcis* x *Prunus persica*), which adapts well to edaphic and climatic conditions, although it is susceptible to *Meloidogyne* spp. Intensive and high-density plantations are gaining ground because of the high yields and cost reduction, as well as organic almond groves, due to growing consumer demand for sustainable agriculture and food safety. Integrated management based on pest identification, progress monitoring, and agronomic practices, such as efficient use of water, cover crops, rootstocks, and biological control, is essential to ensure high yields and economic and environmental sustainability of the crop in the long term.

Plants live in close association with the microorganisms that inhabit the soil, which make up the plant microbiota (Trivedi et al., 2020). The rhizosphere is a natural reservoir of microorganisms, some of which can act as biological control agents (BCAs) by protecting the plant directly or indirectly against pathogens (Köhl et al., 2019). Bacteria, collembola, fungi, predatory nematodes, mites, and protozoans have been reported as BCAs against PPNs, with bacteria and fungi considered to be the most extended and effective nematode antagonists (Stirling, 2014). Some genera of rhizospheric bacteria, such as *Pseudomonas*, *Bacillus*, and *Pasteuria*, have shown efficacy for PPN control (Chen and Dickson, 1998; Abd-El-Khair et al., 2019; Migunova and Sasanelli, 2021). Likewise, nematophagous fungi, such as *Arthrobotrys* spp., *Trichoderma* spp., *Hirsutella rhossiliensis*, *Pochonia chlamydosporia*, and *Purpureocillium lilacinum*, are well-known BCAs against nematodes (Kiewnick and Sikora, 2006; Zhang et al., 2006; Singh et al., 2007; Escudero and Lopez-Llorca, 2012; Pocurull et al., 2020). Additionally, of these specific BCAs against plant-parasitic nematodes, the rhizospheric microbiome plays an important role in plant health. Recent studies have showed that transplanting the rhizospheric microbiome from one plant to another significantly alleviated *M. incognita* and *Pratylenchus penetrans* infections (Elhady et al., 2018; Zhou et al., 2019). However, many of these interactions were difficult to study because the majority of the soil microbiota are difficult or impossible to culture using laboratory methodology. In recent years, massive parallel sequencing has allowed us to increase our knowledge of the soil microbiome. Specifically, several studies have analyzed the occurrence of taxonomic groups of bacteria and fungi, including BCAs, in nematode-infested soils and different crops, such as potato, banana, soybean, and tomato (Castillo et al., 2017; Toju and Tanaka, 2019; Zhou et al., 2019; Ciancio et al., 2022). Since intensive production practices can decrease the occurrence of BCAs, integrated pest management practices and soil microbiota should be considered to increase the efficacy and persistence of beneficial microorganisms (Abd-Elgawad, 2021). Recent studies of the response of soil microbiota in almond crops under different management practices showed changes in the microbial community (Özbolat et al., 2023; Camacho-Sanchez et al., 2023). However, the knowledge of the microbiota associated with almond crop, as well as the interaction of *Meloidogyne* spp. with the almond rhizosphere microbiota and native BCAs, is still limited. Moreover, the severity of damage caused by *Meloidogyne* spp. depends on several factors, including crop, season, soil type, and initial inoculum density (Jones et al., 2013). Thus, knowledge of soil and rhizosphere microbiota associated with almond crops, as well as their interaction with *Meloidogyne* spp., is essential to establish sustainable pest management strategies. The main hypothesis of this research is to know if the increase in root-knot nematode egg levels (and nodulation-root damage) in roots changes the soil ecology, including potential *Meloidogyne* biocontrol agents, in a susceptible hybrid rootstock in commercial almond plantations. Specifically, we analyzed soil and rhizosphere microbial and nematode communities and BCAs associated with the susceptible *Prunus* rootstock (GF-677) in a *Meloidogyne* gradient infection in almond groves. Therefore, six commercial groves with different *Meloidogyne* population densities were selected in order to (i) characterize fungal and bacterial communities of a hybrid rootstock (GF-677) using a metabarcoding approach; (ii) determine the presence of potential indigenous BCAs; (iii) evaluate the effect of different infection root levels of *Meloidogyne* on soil (soil close to the roots), rhizosphere microbiota (soil attached to the roots), and BCAs; and (iv) compare nematode communities between different levels of *Meloidogyne* spp. root infected in different areas of Southern Spain.

## 2 Materials and methods

### 2.1 Study area and sample collection

In a previous survey, the PPN community associated with the rhizosphere of *Prunus* rootstocks was studied in the main stone-production areas in Spain (Clavero-Camacho et al., 2022, 2024). From this study, the groves where selected based on the presence of *Meloidogyne* spp. and with different infection levels throughout the field. Six commercial almond groves in which a *Meloidogyne* root gradient infection was detected in that previous study were considered (all of them in Andalusia, Southern Spain) (**Supplementary Figure 1**). These commercial groves are managed with similar agronomic practices, specifically irrigation, tilling, herbicide application, and the same rootstock [GF-677, susceptible *Prunus* rootstock to *Meloidogyne* spp (Esmenjaud et al., 1997)]. To study different *Meloidogyne* population levels, two to three samples were collected in each grove resulting in a total of 16 sampling points (**Table 1**). Soil and root samples were collected from the rhizosphere of GF-677 rootstock using a shovel and considering the upper 5- to 40-cm depth of soil from four to five trees randomly selected and mixed to constitute a sampling point in each sampling site. The samples were put into polythene bags, transported in coolers to the laboratory, and stored at 4°C until processed. Roots were separated from the soil and divided into two subsamples, one for the extraction of root-knot nematodes (*Meloidogyne* spp.) and the other for rhizosphere microbiota extraction. Homogenized soil was divided into three subsamples, one of which was used for the nematode extraction, another for the physicochemical properties analysis, and the other was frozen at −30°C for total DNA extraction directly from soil (soil microbiota).

**Table 1:** Nematode population density (nematodes per gram of root) and prevalence (%) of root-knot nematodes (*Meloidogyne* spp.) found parasitizing roots of almond in Andalusia (southern Spain).

Sample code	Almond groves	Locality			*Meloidogyne* spp./g of root		
			Lat	Lon	*M. arenaria*	*M. incognita*	*M. javanica*
PR13	1	Córdoba	37.82357778	−4.88607530	-^c^	–	1,209
PR15	1	Córdoba	37.82357778	−4.88607530	–	–	465
PR25A	2	Carmona (Sevilla)	37.51301330	−5.74003634	–	508	–
PR25B	2	Carmona (Sevilla)	37.51301330	−5.74003634	–	639	–
PR25C	2	Carmona (Sevilla)	37.51301330	−5.74003634	–	303	–
PR61A	3	Los Palacios (Sevilla)	37.14548089	−5.86129138	–	220	–
PR61C	3	Los Palacios (Sevilla)	37.14548089	−5.86129138	–	446	–
PR74A	4	Marmolejo (Jaén)	38.01327995	−4.19168131	139	139	–
PR77A	4	Marmolejo (Jaén)	38.02284420	−4.20999267	1,113	–	–
PR78B	4	Marmolejo (Jaén)	38.02078401	−4.17746518	–	448	448
PR90A	5	Alcalá del Río (Sevilla)	37.51437691	−5.97160655	–	–	24
PR90B	5	Alcalá del Río (Sevilla)	37.51437691	−5.97160655	–	–	2,052
PR90E	5	Alcalá del Río (Sevilla)	37.51437691	−5.97160655	–	–	265
PR231B	6	Alcolea (Córdoba)	37.93704798	−4.659543894	–	4,127	–
PR233A	6	Alcolea (Córdoba)	37.93704798	−4.659543894	–	3,588	–
PR234	6	Alcolea (Córdoba)	3.793968296	−4.652517177	–	449	–
**Global**							
**Prevalence^a^**					12.50	62.50	37.5
**Density^b^**					626 (139–1,113)	1,087 (139–4,127)	744 (24–2,052)

a Prevalence = percentage of samples in which a *Meloidogyne* spp. was detected with respect to the total number of samples processed.

b Nematode density = mean of nematodes of each species per gram of root in all sampling points where that genus was detected (mean, minimum, and maximum).

c −: Not detected.

### 2.2 Nematode extraction and identification

Total soil nematodes were extracted from 500-cm³ subsamples of soil by the centrifugal–flotation method (Coolen, 1979) and visualized at ×10–×20 or ×40–×100 magnification. To assess nematode density and prevalence, nematodes extracted from soil were counted and identified at genus level using an integrative taxonomic approach as described by Clavero-Camacho et al. (2024). Then, nematodes were classified into five trophic groups (bacterivores, fungivores, herbivores, omnivores, and predators) according to Yeates et al. (1993). Prevalence was computed by dividing the number of samples in which a nematode genus was detected by the total number of samples and expressed as percentage (Boag, 1993). For each identified genus, nematode density was calculated as the number of individuals of a particular nematode genus per 500 cm³ of soil (Boag, 1993). Two ecological indices [maturity index (MI) for free-living nematodes and plant-parasitic index (PPI)] and three functional indices [channel index (CI), enrichment index (EI), and structure index (SI)] were calculated using the web application Nematode Join Indicator Analysis (NINJA) (Sieriebriennikov et al., 2014) and analyzed with R software 4.3.1 (R Core Team, 2023). After identification and counting, the nematode suspensions were allowed to settle to reduce the volume and frozen at −80°C prior to total DNA extraction for detection of target nematophagous fungi by real-time qPCR.

Regarding RKN, females were collected directly from galled almond roots, while males, second-stage juveniles (J2), and eggs were extracted from roots by blender maceration in a 1% sodium hypochlorite solution (Hussey and Barker, 1973) followed by centrifugal–flotation method (Coolen, 1979). The molecular identification of *Meloidogyne* species was conducted using a multiplex PCR assay from 10 individuals per sample extracted separately in different nematode stages (females, males, and juveniles), which allows the identification of the three most predominant species, specifically, *M. arenaria*, *M. incognita*, and *M. javanica* (Kiewnick et al., 2013) and allowed us to detect species mixtures in the groves. Specifically, multiplex PCR was performed with species-specific primers as follows: Far (5′-TCGGCGATAGAGGTAAATGAC-3′) and Rar (5′-TCGGCGATAGACACTACAACT-3′) (Zijlstra et al., 2000) for *M. arenaria*, Mi2F4 (5′-ATGAAGCTAAGACTTTGGGCT-3′) and Mi1R1 (5′-TCCCGCTACACCCTCAACTTC-3′) (Kiewnick et al., 2013) for *M. incognita*, and Fjav (5′-GGTGCGCGATTGAACTGAGC-3′) and Rjav (5′-CAGGCCCTTCAGTGGAACTATAC-3′) (Zijlstra et al., 2000) for *M. javanica*. The multiplex PCR cycling profile consisted of 15 min 95°C and 40 cycles of 30 s at 94°C, 1 min at 57°C and 2 min at 68°C, with a final extension cycle of 9 min at 68°C. Amplifications were carried out in a final volume of 20 µl using primer concentrations as described by Kiewnick et al. (2013). RKN density in root was calculated as the number of individuals of each *Meloidogyne* species per gram of root. To study the effect of different infection root levels of *Meloidogyne* on soil and rhizosphere microbial communities, RKN density in the roots was categorized into two categories: low (<600 eggs per g of root) and high (>600 egg per g of root). In addition, *Meloidogyne* eggs extracted from roots were used to evaluate fungal egg parasitism.

### 2.3 Analysis of physicochemical soil properties

Prior to physicochemical analysis, the rhizosphere soil was air dried and sieved (2-mm mesh size). These analyses were conducted by the Agri-food Laboratory from Córdoba (Córdoba, Spain). Cation exchange capacity (CEC) was determined using the ammonium saturation method (Bower et al., 1952). The concentration of calcium (Ca) and magnesium (Mg) was measured using the titration method (Diehl et al., 1950). The concentration of sodium (Na) and potassium (K) were determined using the flame photometer method (Richards, 1954). The relative proportion of sand, silt, and clay particles was determined by the Bouyoucos method (Day, 1965), and these data were used to estimate texture according to the USDA soil texture classification. Available phosphorus (P) was measured using the Olsen method (Olsen, 1954). The carbonate content was determined by volumetric method (Allison and Moodie, 1965). Electrical conductivity (EC) and pH of soil were measured in a 1:5 and 1:2.5 soil/water extract, respectively, using a conductivity/pH meter. The percentage of organic matter (OM) was determined by potassium dichromate oxidation (Jackson, 1958) and organic nitrogen content by the Kjeldahl digestion (Bremner, 1965). With these data, the carbon-to-nitrogen (C:N) ratio was calculated. Differences of physicochemical measurements between the six almond groves were tested with ANOVA, followed by Tukey’s honest significant difference (HSD) at p = 0.05.

### 2.4 DNA extraction and quantification

To characterize the fungal and bacterial community associated with *Meloidogyne*-infected almond groves, total genomic DNA was extracted from soil nematodes, soil (soil associated with roots from rhizosphere), and rhizosphere samples (soil and microbes attached to roots and extracted using a specific protocol below). All DNA extractions were performed using DNeasy PowerSoil Pro Kit (Qiagen) according to the manufacturer’s instructions, except that soil DNA was extracted from 500 mg instead of 250 mg. Prior to the rhizosphere DNA extraction, we followed the protocol described by Aranda et al. (2011) to obtain from roots a suspension that includes bacteria and fungi from the rhizoplane adhered soil. Subsequently, 3 ml of each rhizosphere suspension was centrifuged at 11,000 rpm for 4 min and the recovered precipitate was used for rhizosphere DNA extraction. Total DNA from nematodes with adhering microorganisms was extracted using DNeasy PowerSoil Pro Kit (Qiagen) and the FastPrep-24 Instrument (MP Biomedicals, Inc. France) for 40 s at high speed to efficiently disrupt bacterial and fungal cells. All DNA samples were quantified using a Qubit dsDNA HS Assay Kit (Thermo Fisher Scientific) and stored at −20°C until metabarcoding and real-time qPCR analysis.

### 2.5 Fungal egg parasitism

To assess the occurrence and isolate *Meloidogyne* spp. fungal egg parasites, we used the protocol described by Giné et al. (2016) with some modifications. Egg masses could not be handpicked because of the hardness of the woody roots of *Prunus*. In total, 100 µl of eggs’ suspension extracted as mentioned above were spread onto Petri dishes (9-cm diameter) that contain a growth-restricted medium (1% agar; Rose Bengal 50 mg L^−1^; chloramphenicol, 50 mg L^−1^; chlortetracycline, 50 mg L^−1^; streptomycin, 50 mg L^−1^; Triton, 50 mg L^−1^). For each sample, three replicated Petri dishes were prepared with approximately 1,000 eggs per plate. Petri dishes were incubated at 25°C in darkness, and parasitism was evaluated after 48 h under a Leica DMi1 inverted microscope. In each Petri dish, 100 eggs were randomly selected for parasitism visual classification, and the number of parasitized eggs (with fungal hyphae inside) was counted and expressed as percentage of parasitism. At least 20 parasitized eggs per sampling points were individually transferred to potato dextrose agar (PDA) to establish pure cultures. Plates were incubated at 25°C in darkness for 3 to 5 days. Subsequently, morphological and molecular identification of the isolates were performed. Fungal isolates were identified by PCR amplification and sequencing of the ITS region (ITS1-5.8S-ITS2). Fungal DNA extraction was carried out using i-genomic plant DNA extraction mini kit (Intron Biotechnology). The ITS1-5.8S-ITS2 rDNA was amplified using primers ITS5 (5′-GGAAGTAAAAGTCGTAACAAGG-3′) and ITS4 (5′-TCCTCCGCTTATTAGATATGC-3′) (White et al., 1990). The PCR cycling conditions were as follows: 95°C for 15 min, followed by 35 cycles of 94°C for 30 s, 52°C for 30 s, and 68°C for 1 min, and a final extension cycle of 68°C for 7 min. PCRs were performed in a final volume of 20 µl, containing 3 µl of 5× HOT FIREpol Blend Master Mix (Solis Biodyne, Tartu, Estonia), 0.3 µM of each primer, 2 µl of template DNA, and 13.8 µl of ultrapure water. After amplification, PCR products were purified using ExoSAP-IT (Affimetrix, USB products, Kan-del, Germany) and used for direct sequencing in both directions. The resulting products were purified and run in a DNA multi-capillary sequencer (Model 3130XL Genetic Analyzer; Applied Biosystems, Foster City, CA, USA) using the BigDye Terminator Sequencing Kit v.3.1 (Applied Biosystems) at the Stab Vida sequencing facility (Caparica, Portugal). Species identification was implemented by comparison of DNA sequence data obtained in this study with those available in the GenBank database using the basic local alignment search tool (BLAST) at the National Center for Biotechnology Information (NCBI).

### 2.6 Identification of target nematophagous fungi by real-time qPCR

DNA extracted from nematode samples were used to study the occurrence of target organisms by real-time qPCR. A total of six nematophagous fungi (NF) belonging to the three main NF groups (Nordbring-Hertz et al., 2011) and with a different way of nematode parasitism were screened: nematode-trapping (*Arthrobotrys dactyloides*, *A. oligospora*, and *Gamsylella gephyropagum*), endoparasitic (*Catenaria* sp. and *Hirsutella rhossilliensis*), and egg-parasitic fungi (*Pupureocillium lilacinum*). qPCR assays were performed with species-specific primers and Taqman probes. All real-time qPCR assays were performed on an CFX Connect Real Time PCR System (Bio-Rad Laboratories, Inc.) in hard-shell PCR plates 96-well, thin well (Bio-Rad Laboratories, Inc.). These target organisms were detected in independent runs with negative (no sample) controls in all plates, with three technical repetitions per sample. Reactions were performed in a final volume of 20 µl and contained 1× iTaq Universal Probes Supermix (Bio-Rad Laboratories, Inc.), 300 nM BSA (New England BioLabs, Inc.), and 2 ng of DNA per reaction. The specific primers and probes used in this study are shown in **Table 2**. Primer and probe concentrations as well as qPCR conditions were as described by Atkins et al. (2005); Pathak et al. (2012); Campos-Herrera et al. (2019), and Zhang et al. (2006). *Purpureocillium lilacinum* and *Arthrobotrys dactyloides* had been previously isolated and maintained in pure cultures in PDA (Difco Laboratories) at 25°C in darkness. Thus, these fungi could be quantified by real-time qPCR with a standard curve. To do this, DNA was extracted from these pure cultures with i-genomic plant DNA extraction mini kit (Intron Biotechnology) and used to prepare the standard curves. To develop the standard curve, the DNA was diluted in serial 10-fold dilutions from 0.01 to 10^−6^ ng µl^−1^ concentration. For quantification, individual reactions per species were carried out including the standard curve (5 points) and a negative control, each sample per triplicate, following MIQE guidelines (Bustin et al., 2009). For the rest of the target fungi, only their occurrence was studied.

**Table 2:** Specific primers and Taqman probes used to detect/quantify nematophagous fungi in nematode samples.

Organism	Sequence primers and probe (5′–3′)	References for primers/probe concentration and qPCR conditions
*Arthrobotrys dactyloides*	F: AGGTCGGTTTTGAGCTGGCTTA	Pathak et al., 2012; Campos-Herrera et al., 2019
	R: CCACCCCACCTAGAACAAGTATGT	
	P: FAM-ACCCAAGCCGGTTTTAAAGT-MGB	
*Arthrobotrys oligospora*	F: CGGTTTGCTGTTGCAGCTTGTT	Pathak et al., 2012; Campos-Herrera et al., 2019
	R: GGTTCACAAAGGGTTTACCAGG	
	P: FAM-CTGTCTTCCGGTTGGTAAGC-MGB	
*Catenaria* sp.	F: GCCGTGTAGGCAAAAATTCCGACT	Pathak et al., 2012; Campos-Herrera et al., 2019
	R: GCAGCCTGGATTGTTTGATGGCCT	
	P: FAM-TGCTCAACGTCACGAGTAAACCAACA-MGB	
*Hirsutella rhossiliensis*	F419: TGCGCAGTAGCTCCCAGAG	Zhang et al., 2006; Campos-Herrera et al., 2019
	R480: TTGTTTTACGGCGTGACCG	
	P442: FAM-TCGCACCGGAAACGCGGAG-TAMRA	
*Gamsylella gephyropagum*	F: GTCGTAACAAGGTTTCCGTAGG	Pathak et al., 2012
	R: TTGTAAAATGGGTGCCAGCG	
	P: FAM-CCAAAACATAGCTGTCGGGT-MGB	
*Purpureocillium lilacinum*	PLrtF: GACCCAAAACTCTTTTTGCATTACG	Atkins et al., 2005
	PLrtR: AGATCCGTTGTTGAAAGTTTTGATTCATTTGTTTTG	
	PLrt P: FAM-CCGGCGGAATTTCTTCTCTGAGTTGC-TAMRA	

(F, forward primer; R, reverse primer; P, probe).

### 2.7 Bacterial and fungal quantification

Soil and rhizosphere associated with rootstock GF-677 were evaluated for DNA copy number of bacterial/fungi ratio using real-time qPCR following the bacterial protocol and primer concentrations of Jorgensen et al. (2012) with the primers 341F (5′-CCTACGGGNGGCWGCAG-3′) (Herlemann et al., 2011) and 519R (5′-GWATTACCGCGGCKGCTG-3′) (Engelbrektson et al., 2010) in the 16S rRNA and the fungal protocol and primer concentrations of Ihrmark et al. (2012) with the primers gITS7 (5′-GTGAATCATCGAATCTTTG-3′) (Ihrmark et al., 2012) and ITS4 (5′-TCCTCCGCTTA TTGATATGC-3′) (White et al., 1990) in the ITS2 region. Real-time qPCR assays were conducted in polypropylene 96-well plates on an CFX Connect Real Time qPCR System (Bio-Rad Laboratories, Inc.). iQ™ SYBR^®^ Green Supermix (Bio-Rad Laboratories, Inc.) with 5 ng of DNA/reaction in a final volume of 20 µl. Each sample was quantified in a triplicate reaction. Control samples without DNA template (NTC) were included in each plate in triplicate. Bovine serum albumin (BSA) (New England BioLabs, Inc.) was added at a final concentration of 300 nM to reduce the potential inhibition of humic acids and other compounds present in soil (Kreader, 1996). Melting curve analysis of the PCR products was conducted following each assay to confirm that the fluorescence signal originated from specific PCR products and not from primer dimers or other artifacts. Standard curves were constructed using purified PCR products from the 16S rRNA of *Escherichia coli* using the primers 8F and 1492R (Turner et al., 1999) and ITS products from *Rosellinia necatrix* using the primers ITS1f (Gardes and Bruns, 1993) and ITS4. PCR products were purified using GeneClean^®^ Turbo Kit (MP Biomedicals), and the molecular weight of each purified DNA fragment was calculated based on the number of bases of the DNA fragment. Estimation of gene-copy number in each representative purified DNA fragment was based on the molecular weight in accordance with DNA Copy Number and Dilution Calculator (ThermoFisher Scientific), with copy number adjusted in a 10-fold dilution series used to generate standard curves for real-time experiments. The calibration curves were linear over eight orders of magnitude (100–10^6^). Optical data was visualized and analyzed in CFX Maestro™ Software v. 1.1 (Bio-Rad Laboratories, Inc.). Differences of fungal:bacterial ratio, ITS and 16S copy numbers between sample type (rhizosphere, soil) and between *Meloidogyne* density (low, high) were evaluated (Wilcoxon, p < 0.05).

### 2.8 DNA metabarcoding library preparation and sequencing

Rhizosphere and soil samples were analyzed to study the bacterial and fungal diversity using a metabarcoding sequencing approach. DNA metabarcoding analyses were carried out by AllGenetics & Biology S.L. (La Coruña, Spain) (www.allgenetics.eu). The DNA extracted from soil and rhizosphere samples was adjusted to 10 ng µl^−1^ and used to amplify the V3–V4 region of bacterial 16S rRNA gene and the internal transcribed spacer 2 (ITS2) region of fungi. The PCR of the 16S rRNA region was carried out with the primers 341F (5′-CCTACGGGNGGCWGCAG-3′) (Herlemann et al., 2011) and 805R (5′-GACTACHVGGGTATCTAATCC-3′) (Herlemann et al., 2011) to amplify a fragment of approximately 420 bp. The primers ITS86F (5′-GTGAATCATCGAATCTTTGAA-3′) (Turenne et al., 1999) and ITS4 (5′-TCCTCCGCTTATTGATATGC-3′) (White et al., 1990) were used to amplify the complete fungal ITS2 region of approximately 300 bp. All primers were modified to include Illumina adapters to their 5′ ends. To prevent the amplification of the host *Prunus* DNA, a blocking primer was included in the library preparation. The blocking primer BP_Bakt805R_prunus (5′-CTAATCCCATTTGCTCCCCTAGCTTTCGTCT-3′) was previously designed following the approach of Vestheim and Jarman (2008) with *Prunus* sp. chloroplast 16S sequences deposited in GenBank and using Geneious 11.1.5. Blocking primers were modified with a C3 (3 hydrocarbons) at the 3′ end to prevent elongation of *Prunus* sp.

For bacteria, PCRs were performed in a final volume of 12.5 µl containing 1.25 µl of template DNA, 300 nM of the primers, 6 µM of the blocking primers, 3.25 µl of Supreme NZYTaq 2x Green Master Mix (NZYTech), and ultrapure water up to 12.5 µl. The PCR conditions were as follows: 95°C for 5 min, followed by 20 cycles of 95°C for 30 s, 50°C for 45 s, 72°C for 45 s, and a final extension step at 72°C for 7 min. The oligonucleotide indices that are required for multiplexing different libraries in the same sequencing pool were attached in a second amplification step with identical conditions but only 5 cycles and 60°C as the annealing temperature. A negative control with water instead of DNA template was included in every PCR round.

For fungal library preparation, two amplification steps were also performed. First, amplifications of ITS2 region were carried out in a final volume of 12.5 µl, containing 3.25 µl of template DNA, 0.5 µM of each primer, 10 µM of the blocking primers, 3.25 µl of Supreme NZYTaq 2× Green Master Mix (NZTYech), and ultrapure water up to 12.5 µl. PCR amplifications consisted of a 5-min denaturation at 95°C, followed by 35 cycles of 30 s at 95°C, 45 s at 49°C, 45 s at 72°C, and a final extension of 7 min at 72°C. The oligonucleotide indices that are required for multiplexing different libraries in the same sequencing pool were attached in a second amplification step with identical conditions but only 5 cycles and 60°C as the annealing temperature. Negative control (no template DNA) were included in all PCRs.

To verify the fungal and bacterial libraries size, the PCR products were run on 2% agarose gels stained with GreenSafe (NZYTech). Libraries were purified using the Mag-Bind RXNPure Plus magnetic beads (Omega Biotek) following the manufacturer’s instructions. Then, libraries were quantified using a Qubit dsDNA HS Assay (Thermo Fisher Scientific), and equimolar amounts from each individual sample were pooled and sequenced in a fraction of a NovaSeq PE250 flow cell (Illumina) at the AllGenetics and Biology SL facilities (La Coruña, Spain).

### 2.9 Sequence processing

The quality of the FASTQ files was checked with the software FastQC (Andrews, 2010), and the output was summarized with MultiQC (Ewels et al., 2016). The tool DADA2 (Callahan et al., 2016a), implemented in QIIME2 (Bolyen et al., 2019), was used to trim primers, filter reads according to their quality, denoise using the parametric error model, merge the forward and reverse reads, remove the chimeric reads, and cluster the resulting sequences into amplicon sequence variants (ASVs). In the case of fungi, due to the high length variability of the ITS2 region, together with the fact that the sequencing reads were longer than the amplicons, non-biological DNA (primers or sequencing adapters) could appear at the ends of the reads. Therefore, Cutadapt (Martin, 2011) was used to remove primer and/or adapter sequences. After checking the quality, reads were truncated at position 249 for forward reads, and at position 240 for reverse reads, for bacteria. In the case of fungi, reads were truncated at position 248 for forward reads and at position 249 for reverse reads. After processing the reads with DADA2, a table with the occurrences of each observed ASV in each sample was created. The taxonomic assignment of each ASV was conducted using a pre-trained classifier of the SILVA reference database (Quast et al., 2012) for bacteria, and the UNITE v8.3 reference database (Abarenkov et al., 2020) for fungi, using, in both cases, the *feature-classifier classify-sklearn* approach implemented in QIIME2 (Bokulich et al., 2018). A final OUT table for each combination of target region (ITS2 or 16S) and sample type (soil or rhizosphere) was generated excluding Singletons, eukaryotic sequences of chloroplast and mitochondrial origin, unidentified sequences, ASVs occurring at a frequency below 0.01% in each sample, and sequences assigned only at kingdom level. The final filtered ASV table was converted into a Biological Observation Matrix file (.biom) that was imported into R software 4.3.1 (R Core Team, 2023) using the *phyloseq* package (v1.44.0) (McMurdie and Holmes, 2013) for further analyses.

To construct the ASV-based phylogenetic trees, ASV sequences after the filtering process were aligned using MAFFT (v7.490) (Katoh and Standley, 2013). The alignment was used to generate a maximum likelihood tree in IQ-TREE (v2.2.0) (Minh et al., 2020) with 1,000 ultrafast bootstrap replicates and the SH-like approximate likelihood ratio test (Guindon et al., 2010). The best-fit models were selected by jModelTest (Darriba et al., 2012) according to the Bayesian information criterion (BIC). The model for the bacterial ASVs was GTR+F+I+G4 and for the fungal ASVs was GTR+F+G4, the GTR model with base frequencies empirically computed from the MSA (+F), with invariant sites allowed, with four categories of site-rate heterogeneity under the Gamma mode. Subsequently, phylogenetic trees were rooted at their midpoint using the midpoint.root function in the *phytools* R package (v1.9.16) (Revell, 2012).

Finally, we synthesized all data generated (ASV table, taxonomy table, phylogenetic tree, and sample data) into one phyloseq object for bacterial ASVs and another object for fungal ASVs, in R using the *phyloseq* package. The sample data set included sample information, such as locality, sample type (soil or rhizosphere), fungal:bacterial ratios, soil physicochemical properties, and abundance data of the identified nematode genera.

Data processing was done in AllGenetics & Biology S.L. (La Coruña, Spain) and the supercomputing platform in High Performance Computing Cluster provided by the Centro Informático Científico de Andalucía, Junta de Andalucía. The original raw sequences have been deposited in the NCBI SRA archive (Bioproject: PRJNA993485).

### 2.10 Fungal and bacterial diversity

After taxonomic assignment of each ASV, we checked that all ASVs were assigned to phylum level and applied prevalence filtering (Callahan et al., 2016b). First, we determined the prevalence, i.e., the fraction of total samples in which an ASV was observed, and defined a prevalence threshold of 6.25% to retain phyla detected in at least two of the samples analyzed. We also removed ASVs for which no class could be assigned. Subsequently, analyses of the composition, diversity, and functions of the microbial communities were performed using R software version 4.3.1.

To explore the composition of the fungal and bacterial communities in soil and rhizosphere, bar plots were generated using the *microbiome* package (1.22.0) (Lahti and Shetty, 2012-2019) to show all phyla, orders, and families with >1% relative abundances, while phyla, orders, and families with <1% relative abundance were grouped as “other.” Alpha diversity measurements, including the number of observed ASVs, Shannon diversity index, and Faith phylogenetic diversity, were calculated using the packages *phyloseq* (1.44.0) and *picante* (1.8.2) (Kembel et al., 2010). Statistical significance of differences in diversity estimates in soil and rhizosphere were tested by Wilcoxon rank-sum tests, and data were not transformed. Principal coordinate analysis of Bray–Curtis and weighted Unifrac distance matrices were used to evaluated differences among microbial communities according to sample type (soil or rhizosphere). To understand the relationship between fungal and bacterial communities in rhizosphere and soil, PCoA plots were generated using the ordinate and plot_ordination functions in *phyloseq*, and data were not transformed. To test the effect of sample type in community composition, Bray–Curtis and weighted Unifrac distance matrices were subject to permutational analysis of variance (PERMANOVA) with 999 random permutations using the adonis2 function within the *vegan* package (2.6.4) (Oksanen et al., 2007). We chose this statistical analysis because it is a powerful statistical technique to detect changes in community structure (Anderson and Walsh, 2013). Analysis of the homogeneity of variance among sample type groups was performed using the betadisper function in the *vegan* package. According to the relative abundance of genera in the fungal and bacterial community compositions, the top ranked 25 genera were selected to construct a clustering heatmap. Heatmaps were performed using the *microbiomeutilities* package (1.0.17) (Shetty and Lahti, 2023).

The linear discriminant analysis effect size (LEfSe) algorithm (Segata et al., 2011) was carried out to identify taxa, at genus level or above, that significantly differed between low and high *Meloidogyne* densities in roots. Rhizosphere and soil samples were analyzed separately. Analyses were performed using the *lefser* package (1.10.1) (Khleborodova, 2023). The threshold for the logarithmic linear discriminant analysis (LDA) score was set at 2.0 and the Wilcoxon p-value at 0.05. Results with p < 0.05 between groups were considered statistically significant and represented in bar plots.

The ecological guild information was extracted for each fungal ASV based on their taxonomic assignment using the FUNGuild database (Nguyen et al., 2016) with the *FUNGuildR* package (0.2.0.9) (Furneaux and Song, 2021). The assignments classified as “probable” and “highly probable” in the confidence ranking were selected and grouped into eight categories based on trophic groups and guilds: animal pathogen, arbuscular mycorrhizal, ectomycorrhizal, endophyte, endophyte-plant pathogen, fungal parasite, plant pathogen, and saprotroph. Regarding the functional analysis of bacterial communities, the software PICRUSt2 (Douglas et al., 2020) was used to perform functional predictions based on 16S rRNA gene sequencing data. Predicted Kegg Ortholog (KO) abundance data was imported into R and converted to KEGG pathway abundances using the *ggpicrust2* package (1.7.3) (Yang et al., 2023). Then, a differential abundance analysis was performed using the ALDEx2 method to determine if microbial communities in soil and rhizosphere samples from almond groves with low *Meloidogyne* densities in roots differed functionally from samples with high *Meloidogyne* densities in the roots. The KEGG pathways were also mapped to primary and secondary pathways. The primary pathways were classified into six fundamental metabolic categories: cellular processes, environmental information processing, genetic information processing, human diseases, metabolism, and organismal systems.

## 3 Results

### 3.1 Nematode community assessment and ecological analysis

**Figure 1:**
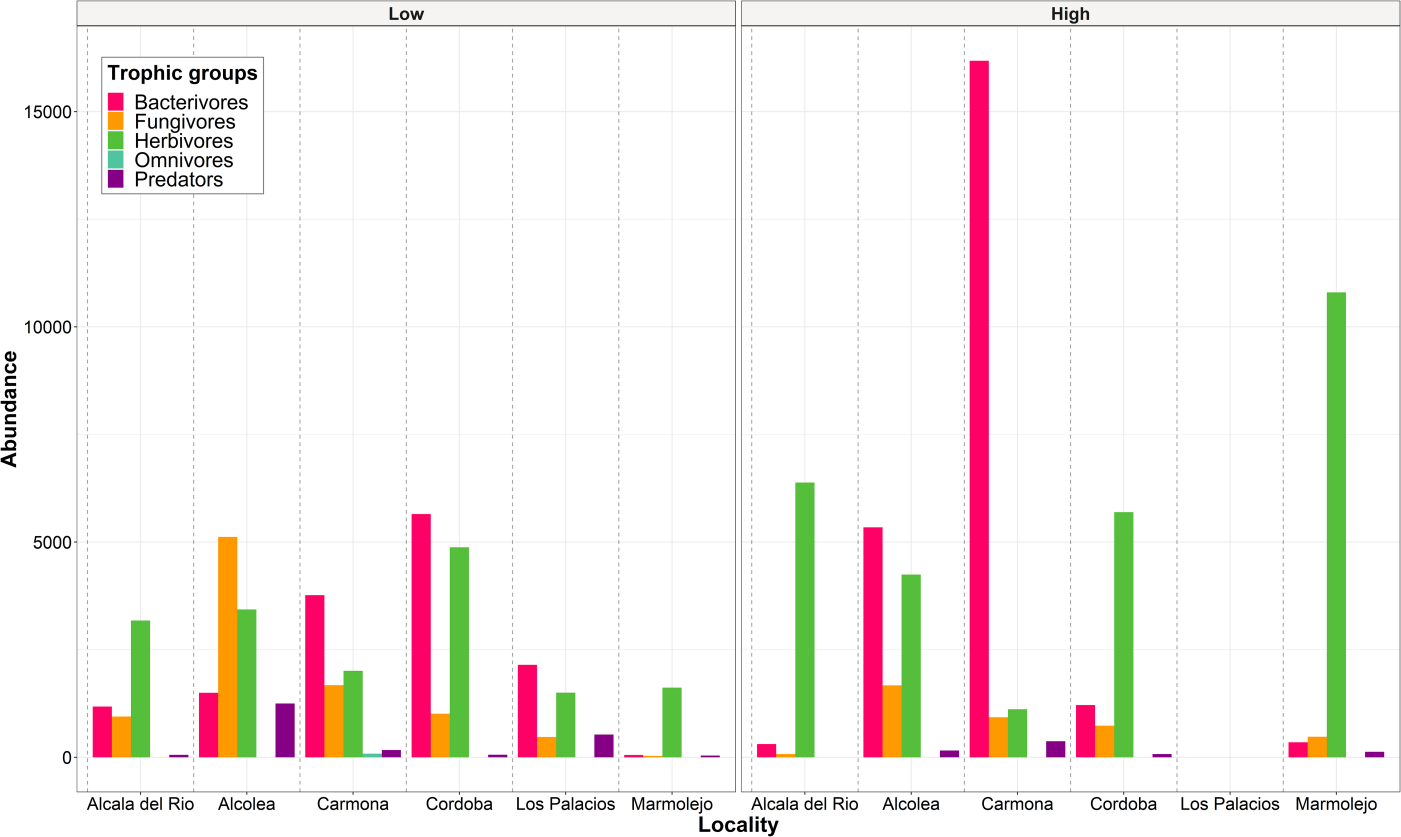
Abundance (number of individuals of each trophic group/500 cm^3^ of soil) of nematode trophic groups in each locality studied separating the samples according to *Meloidogyne* density categories: low (<600 eggs per g of root) and high (>600 eggs per g of root).

A total of 41 nematode genera belonging to 27 families were identified in the rhizosphere of the six commercial almond groves studied (16 samples). The total prevalence and density of these genera are shown in **Supplementary Figure 2** and **Supplementary Table 1**. Nematodes reported in this study belong to five trophic groups, with bacterivores and herbivores being the most diverse, both with 13 genera, followed by fungivores and predators both with 7 genera, and omnivores with only 1 genus. Bacterivores and herbivores were the most abundant trophic groups, followed by fungivores. Specifically, in four of the six almond groves, herbivores were the most abundant, and in the remaining two groves, bacterivores were the most abundant (**Figure 1**). Besides *Meloidogyne* spp., 12 other genera of PPNs (herbivores) were found in almond rhizosphere, including *Paratylenchus*, *Pratylenchus*, and *Xiphinema*. From these 12 genera, *Paratylenchus* was the most prevalent, found in 62.5% of the samples, followed by *Helicotylenchus*, *Merlinius* and *Tylenchorhynchus*, all three in 31.25% of the samples. Within bacterivores, *Cephalobus*, *Mesorhabditis*, *Chiloplacus*, *Acrobeles*, and *Stegelletina* were the most widespread genera, detected in 81.25%, 75.00%, 62.50%, and 56.25% of the samples, respectively. Regarding fungivores, *Filenchus*, *Aphelenchoides*, and *Aphelenchus* were the most prevalent, which were identified in 87.50%, 75.00%, and 56.25% of the samples, respectively. Although *Aporcelaimus*, *Discolaimus*, and *Aporcelaimellus* were present in 56.25%, 31.25%, and 25.00% of the samples, predators, along with omnivores, were the least abundant trophic groups.

**Figure 2:**
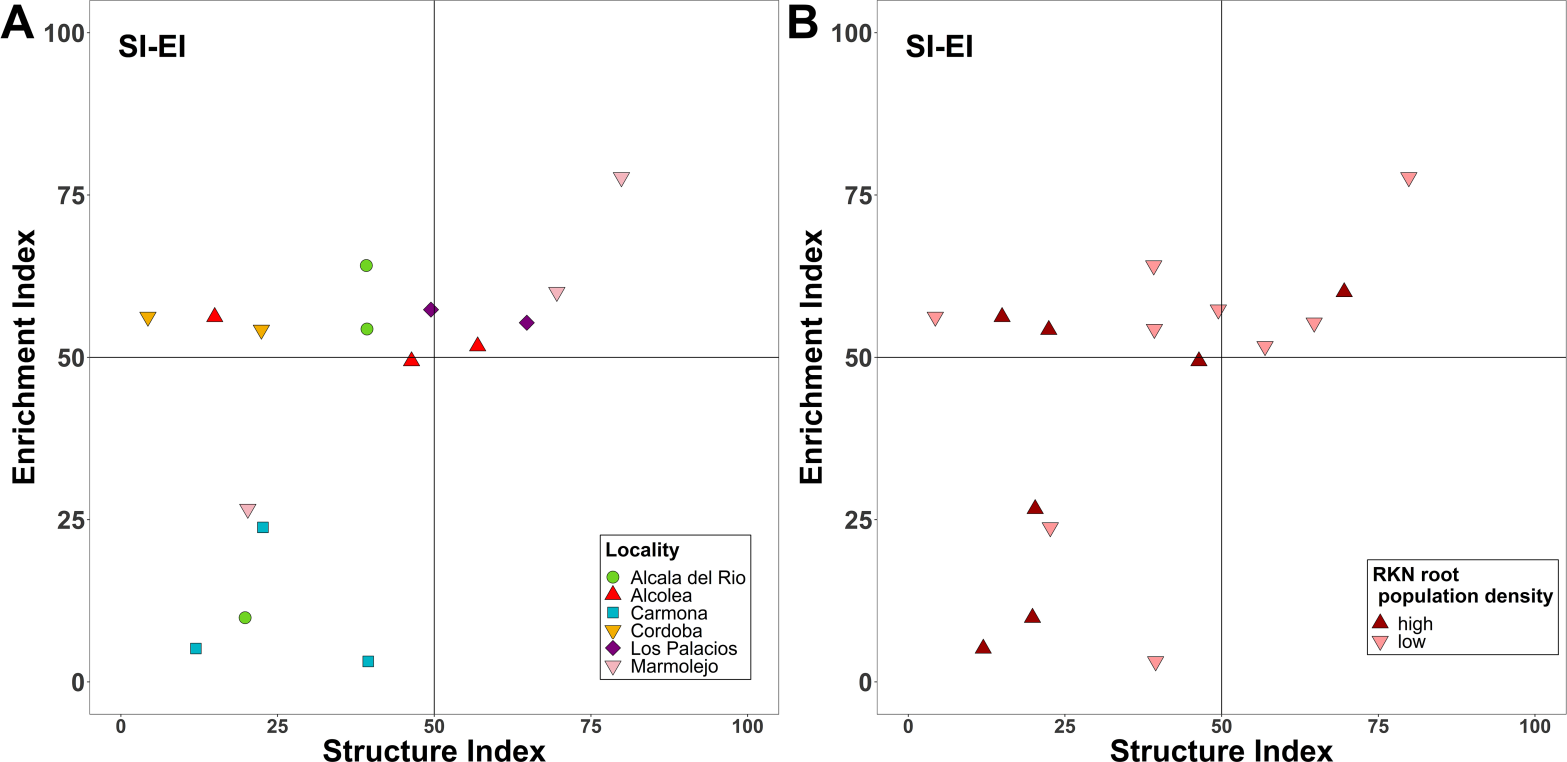
Nematode fauna profiles representing the structure and enrichment conditions of the soil food web for the different localities sampled **(A)** and for the two defined categories of *Meloidogyne* population density in roots [low (<600 eggs per g of root) and high (>600 eggs per g of root)] **(B)**.

The MI and PPI are indicators of the ecological successional status of a soil community. The MI varied from 2.36 to 1.88. No differences (p > 0.05) were found between localities (**Supplementary Figure 3A**). Likewise, PPI did not show significant differences between localities, with values ranging from 3.16 to 2.5 (**Supplementary Figure 3B**). For the CI, significant differences were observed between the commercial almond groves located in Carmona and Los Palacios. The CI, indicators of organic matter decomposition mediated by fungi, ranged from 100% (in Carmona) to 17.78% (in Los Palacios) (**Supplementary Figure 3C**). Neither SI, indicator of soil food web complexity, nor the EI, indicator of organic matter decomposition mediated by bacteria, significantly changed between localities. The EI and SI are descriptors of food web condition; a diagram representing these indices classified soils as maturing, disturbed, and degraded. The majority of the soil samples were in the disturbed N-enriched quadrant (n = 6) and degraded–depleted quadrant (n = 6) and the other samples in the maturing N-enriched quadrant (n = 4) (**Figure 2A**). Only all sampling points were in the quadrant degraded and depleted Carmona field, and all points of Cordoba field were in the disturbed and N-enriched quadrant. Sample distribution in this graph was not associated to *Meloidogyne* density in roots (**Figure 2B**). Additionally, MI, PPI, and CI were not statistically different among the low and high *Meloidogyne* densities in roots (**Supplementary Figure 4**).

### 3.2 Physicochemical soil properties

The commercial almond groves showed statistically significant differences in several of the soil physicochemical properties studied (**Supplementary Table 2**). Soil pH, cation exchange capacity, as well as concentration of calcium, magnesium, potassium, and sodium, were lower in the samples located in Carmona (PR25A–PR25C) than the other sampling groves. Specifically, the soil pH in Carmona was 5.6, while in the rest of the samples, the pH was approximately 8.6. The percentage of organic matter (OM), phosphorous content (P), and carbon to nitrogen (C:N) ratio were similar in the six groves (**Supplementary Table 2**).

### 3.3 Egg-parasitic fungi isolated against *Meloidogyne* spp.

**Figure 3:**
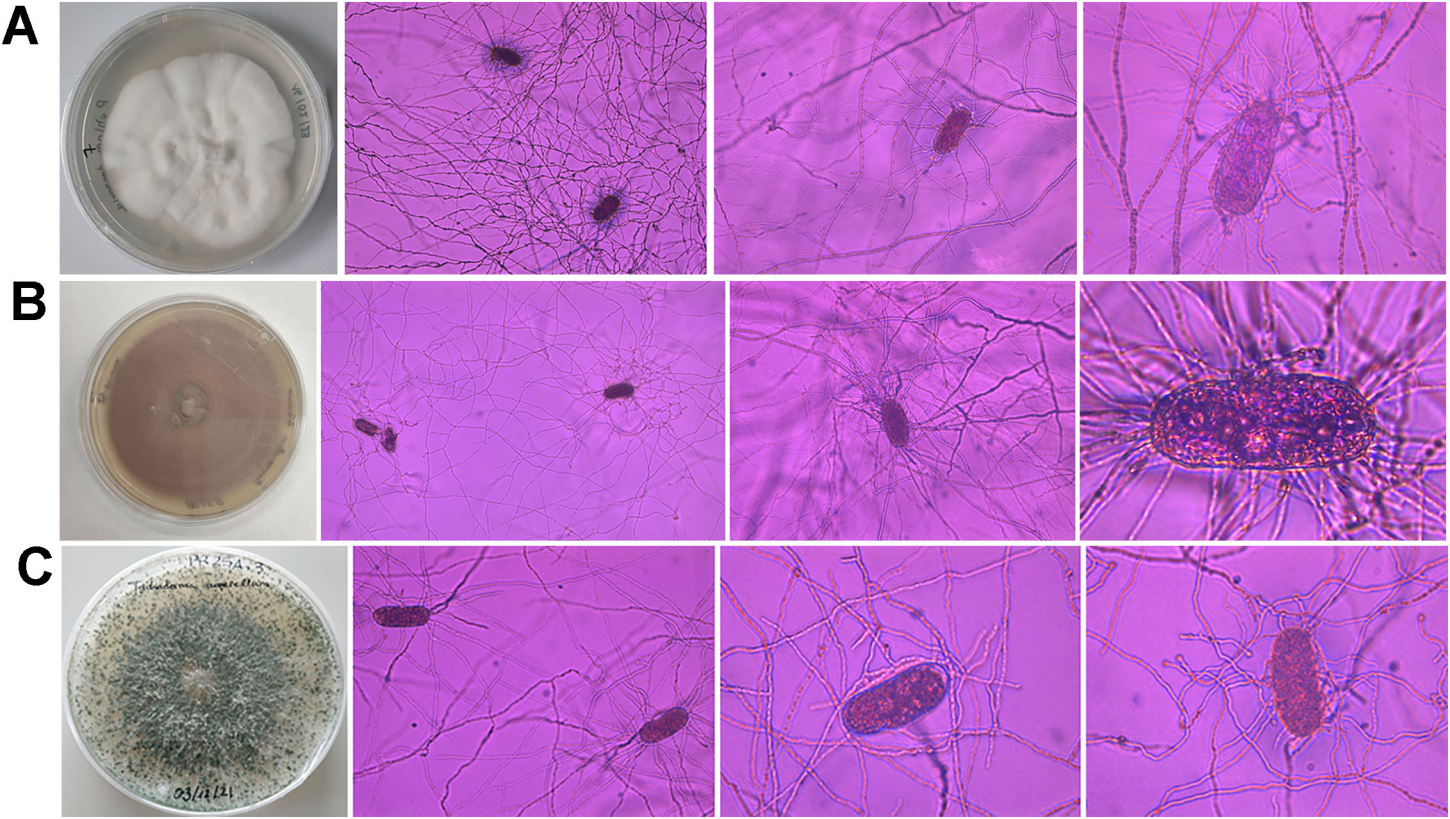
Morphological characteristics of *Pochonia chlamydosporia* **(A)**, *Purpureocillium lilacinum* **(B)**, and *Trichoderma asperellum* **(C)** colonies on PDA and *Meloidogyne* eggs parasitized by these fungi.

After spreading eggs extracted from almond roots in Petri dishes containing a growth-restricting medium, fungal parasites of *Meloidogyne* eggs were found in 56.25% of the samples (**Table 3**). The percentage of parasitized eggs by fungi ranged from 1% to 8%. Three fungal species were isolated from *Meloidogyne* eggs, specifically *Pochonia chlamydosporia*, *Purpureocillium lilacinum*, and *Trichoderma asperellum* (**Figure 3**). All sequences obtained for these species matched well with the accessions from the same species deposited in GenBank, showing 99%–100% similarity. In addition, the morphology of these fungal isolates agreed with that described in literature (Samson, 1974; Samuels, 1996; Evans and Kirk, 2017). The most frequent fungal species isolated was *P. chlamydosporia*, which was found in three of the six commercial almond groves, specifically in Alcala del Rio, Carmona, and Marmolejo, while *P. lilacinum* and *T. asperellum* were only isolated in one grove, both in Carmona. In this study, five *P. chlamydosporia* sequences, four *P. lilacinum* sequences, and seven *T. asperellum* sequences were deposited in the GenBank database (OR801652–OR801667).

**Table 3:** Percentage of parasitized eggs of *Meloidogyne* spp. and samples in which target nematophagous fungi were detected by real-time qPCR.

Sample	Almond grove	Locality	*Fungal egg parasitism (%)*	*Arthrobotrys oligospora*	*Arthrobotrys dactyloides*	*Catenaria* sp.	*Purpureocillium lilacinum*
PR13	1	Córdoba	7	-^a^	–	–	+
PR15	1	Córdoba	8	–	+^b^	+	+
PR25A	2	Carmona (Sevilla)	3	–	–	–	+
PR25B	2	Carmona (Sevilla)	3	–	–	–	+
PR25C	2	Carmona (Sevilla)	3	–	–	–	+
PR61A	3	Los Palacios (Sevilla)	0	+	–	–	+
PR61C	3	Los Palacios (Sevilla)	0	+	–	–	+
PR74A	4	Marmolejo (Jaén)	0	–	–	–	+
PR77A	4	Marmolejo (Jaén)	2	–	–	–	+
PR78B	4	Marmolejo (Jaén)	0	–	–	–	–
PR90A	5	Alcalá del Río (Sevilla)	1	–	+	–	+
PR90B	5	Alcalá del Río (Sevilla)	1	–	–	–	+
PR90E	5	Alcalá del Río (Sevilla)	1	–	–	–	+
PR231B	6	Alcolea (Córdoba)	0	–	–	–	+
PR233A	6	Alcolea (Córdoba)	0	–	–	–	+
PR234	6	Alcolea (Córdoba)	0	–	+	–	+

a −: Not detected.

b +: Detected.

### 3.4 Fungal and bacterial quantification by real-time qPCR

The microbial communities were quantified by real-time qPCR of the 16S rRNA gene and ITS region. Total bacteria were quantitatively similar between rhizosphere and soil samples, with a mean abundance of 1,153,500 ± 453,021 and 1,306,000 ± 460,084 16S rRNA gene copies per 1 ng of DNA, respectively, while the fungal ITS gene copy numbers were significantly higher in rhizosphere samples (54,213 ± 46,634) than in soil samples (12,266 ± 11,226). Likewise, the fungal:bacterial ratio showed a similar trend; rhizosphere samples had a significantly higher ratio than soil samples (**Supplementary Figure 5**). No significant differences were found when comparing fungal and bacterial quantification data between low and high *Meloidogyne* densities (p > 0.05) (**Supplementary Figure 5**).

### 3.5 Detection of nematophagous fungi by real-time qPCR

Four of the six nematophagous fungi screened were detected in the nematode samples by real-time qPCR as follows: *Arthrobotrys dactyloides*, *A. oligospora*, *Catenaria* sp,. and *Purpureocillium lilacinum* (**Table 3**). *Purpureocillium lilacinum* was the most widespread nematophagous fungi, detected in 15 of the 16 sampling points. *Arthrobotrys dactyloides* was detected in three sampling sites, in three different almond groves, while *A. oligospora* was detected in two sampling points in the same almond grove. *Catenaria* sp. was detected only in one sample.

### 3.6 Metabarcoding analyses

Rarefaction curves of ITS region and 16S rRNA sequencing for all samples were constructed to assess species richness and estimated the sequencing depth (**Supplementary Figure 6**). Rarefaction analysis of 16S rRNA sequences showed that two rhizospheric samples, PR74Ar and PR78Br, did not reach the plateau in species richness (**Supplementary Figure 6C**) and were therefore removed from the ASV table. A total of 1,932,009 and 3,091,470 reads remained for the fungal and bacterial libraries, respectively. For fungal data set, the number of sequences in each sample ranged from 14,709 to 116,075, with an average of 60,375 (standard deviation, *s.d*.: 22,081). After the quality and prevalence filtering steps, a total of 1,194 ASVs for ITS and 8,644 ASVs for 16S were obtained. For the bacterial data set, the number of sequences in each sample ranged from 19,051 to 206,974, with an average of 103,049 (*s.d*.: 28,813).

**Figure 4:**
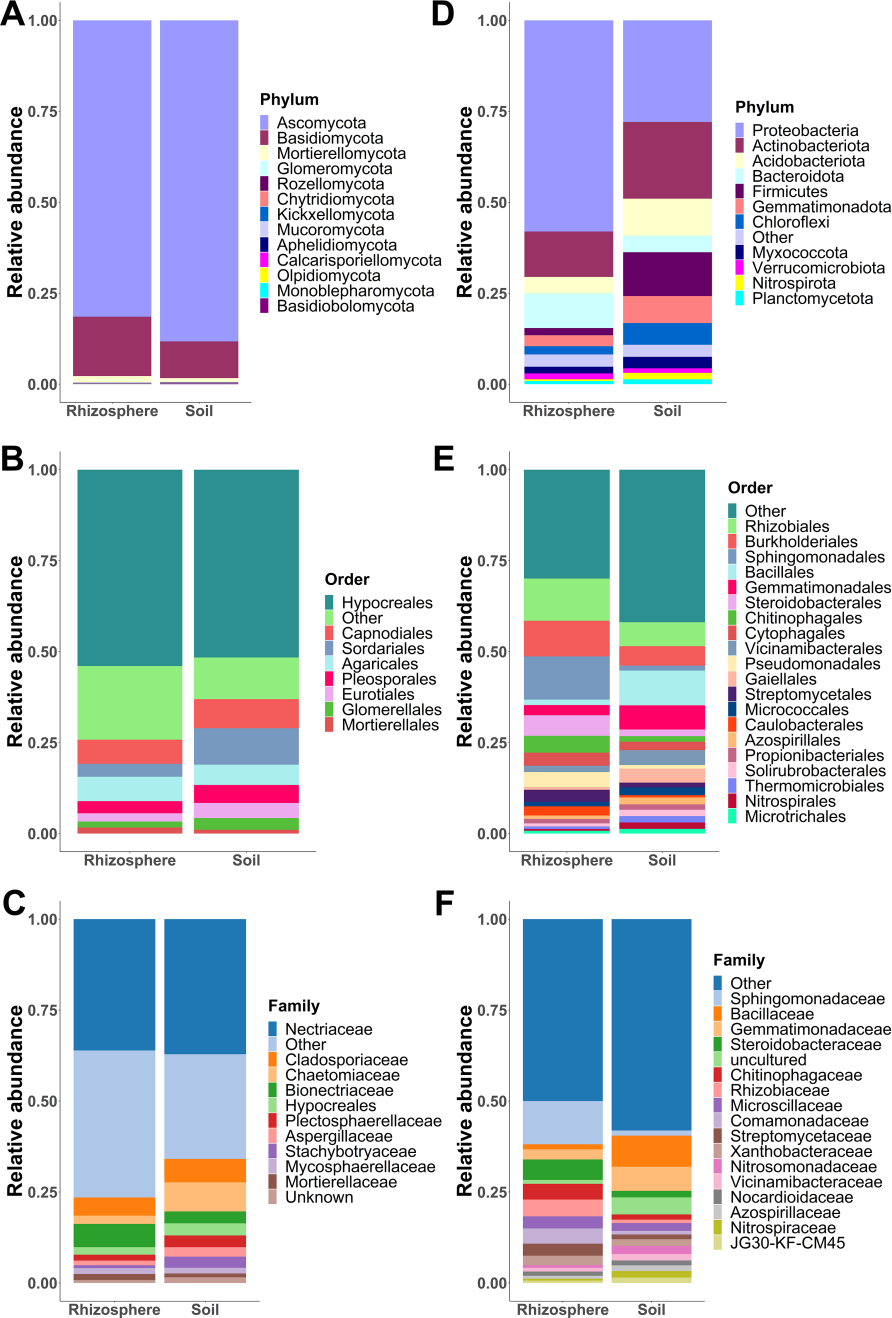
Fungal and bacterial community composition. Relative abundances of fungal phyla **(A)**, orders **(B)**, and families **(C)** pooled by sample type (soil and rhizosphere). Relative abundances of bacterial phyla **(D)**, orders **(E)**, and families **(F)** grouped by sample type (soil and rhizosphere). Phyla, orders, and families with a relative abundance greater than 1% are shown, while those with a relative abundance less than 1% are merged as “others.”.

The 1,194 fungal ASVs were assigned to 13 phyla, 35 classes, 80 orders, 162 families, 301 genera, and 377 species. Ascomycota was the most abundant phylum with a relative abundance of 81.44% and 88.25% in rhizosphere and soil samples, respectively. Basidiomycota was the second phylum most abundant in both rhizosphere (16.31%) and soil (10.06%) samples, while the remaining 11 phyla together accounted for less than 3% of the relative abundance (**Figure 4A**). At the order level, eight orders accounted for almost 90% of the relative abundance, with Hypocreales being the most abundant in both rhizosphere (54.10%) and soil (51.70%) (**Figure 4B**). In the rhizosphere, the next seven most abundant orders were as follows: Agaricales (6.72%), Capnodiales (6.63%), Polyporales (4.29%), Sordariales (3.57%), Branch06 (3.43%), Pleosporales (3.34%), and Eurotiales (2.29%), whereas in soil, the next seven most abundant orders were as follows: Sordariales (10.02%), Capnodiales (8.03%), Agaricales (5.60%), Pleosporales (4.96%), Eurotiales (4.16), Glomerellales (3.23%), and Tremellales (1.70%). In both rhizosphere and soil samples, Nectriaceae was the most dominant fungal family with a relative abundance of 36.43% and 37.65%, respectively (**Figure 4C**). In soil, Chaetomiaceae (8.17%) and Cladosporiaceae (6.56%) were the second and third most abundant families, although the relative abundance of both families decreased in the rhizosphere to 2.31% and 5.04%, respectively. In contrast, the relative abundance of Bionectriaceae increased from 3.30% in the soil to 6.41% in rhizosphere samples (**Figure 4C**).

Regarding bacterial community composition, the 8,644 ASVs were assigned to 36 phyla, 93 classes, 216 orders, 334 families, 609 genera, and 370 species. In both sample types, Proteobacteria and Actinobacteriota were the most abundant phyla (**Figure 4D**). However, the relative abundance of Proteobacteria was higher in the rhizosphere (58.04%) than in the soil (27.94%) in contrast with Actinobacteriota, whose relative abundance was lower in rhizosphere samples (12.47%) than in soil samples (21.06%). Both phyla accounted for around 70% of the relative abundance in the rhizosphere. Firmicutes and Acidobacteriota were the third and fourth most abundant phyla in the soil; however, their relative abundance decreased in the rhizosphere from 12.05% to 1.99%, and from 10.17% to 4.50%, respectively (**Figure 4D**). In the rhizosphere, Sphingomonadales (11.91%), Rhizobiales (11.63%), and Burkholderiales (9.82%) were the most abundant orders. While the three most abundant orders in soil samples were as follows: Bacillales (9.58%), Gemmatimonadales (6.67%), and Rhizobiales (6.61%) (**Figure 4E**). At the family level, Sphingomonadaceae (11.96%) was the most abundant family in rhizosphere samples, followed by Steroidobacteraceae (5.65%), Rhizobiaceae (4.65%), Chitinophagaceae (4.35%), and Comamonadaceae (4.23%). In contrast, these families showed a relative abundance of less than 2% in the soil (**Figure 4F**). In the soil, the most abundant families were as follows: Bacillaceae (8.63%), Gemmatimonadaceae (6.71%), Rubrobacteriaceae (2.25%), Nitrosomonadaceae (2.21%), and Microscillaceae (2.17%) (**Figure 4F**). In both soil and rhizosphere, most orders and families showed a relative abundance of less than 1%, so these were merged into the “other” category for graphical representation of fungal and bacterial community composition.

**Figure 5:**
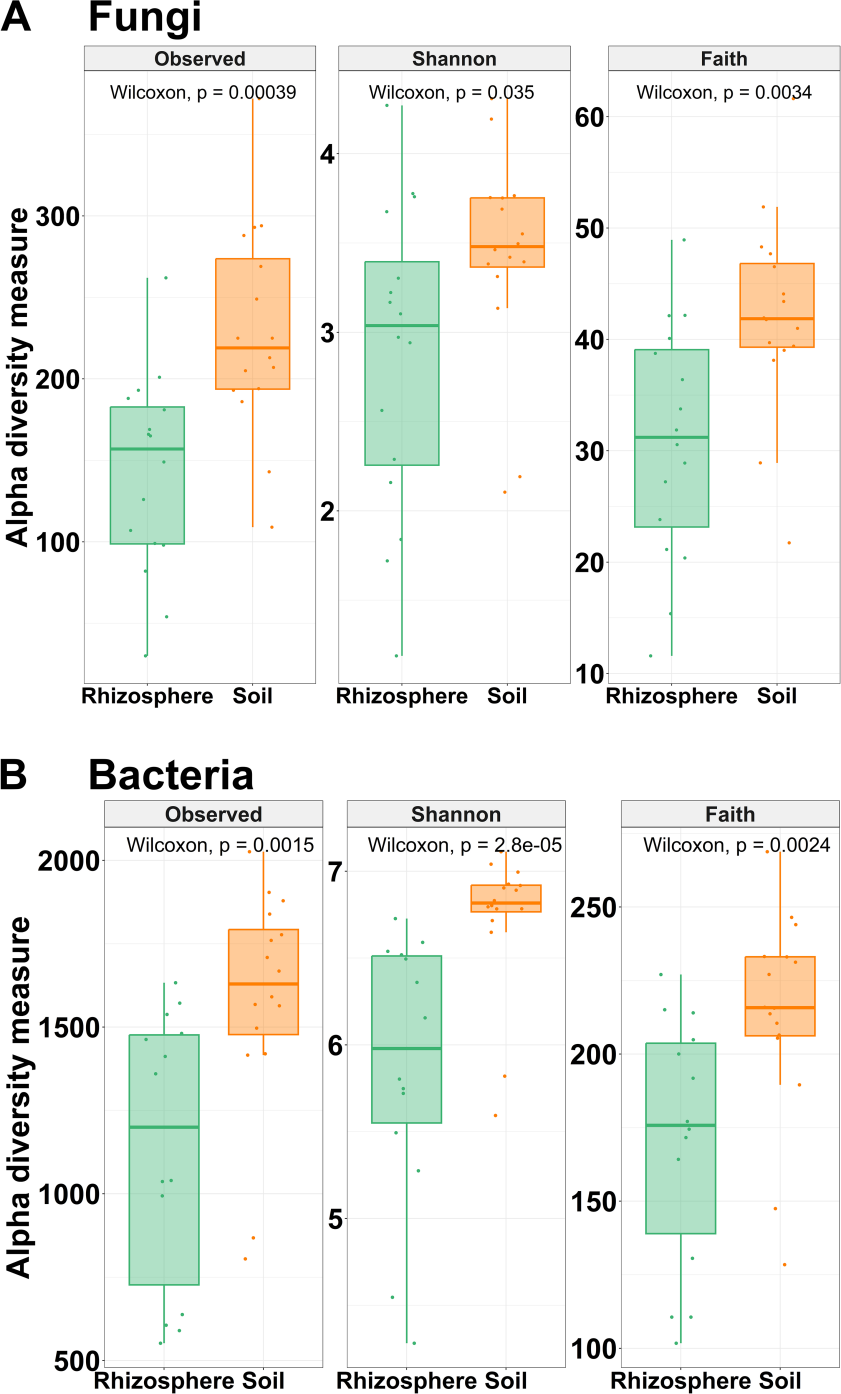
Alpha diversity. Box plots illustrating the differences in the three metrics of alpha diversity (observed ASVs, Shannon diversity index, and Faith’s phylogenetic diversity index) of the fungal **(A)** and bacterial **(B)** communities between soil and rhizosphere samples. Wilcoxon rank-sum tests between soil and rhizosphere samples are shown. All comparisons were statistically significant by Wilcoxon rank-sum tests (*p* < 0.05).

Fungal and bacterial alpha diversity, including observed ASVs, Shannon diversity, and Faith’s phylogenetic diversity (Faith’s PD) indices, decreased significantly (p < 0.05) in rhizosphere samples compared to soil samples (**Figure 5**). Rhizosphere samples have significant lower fungal species richness (142 ± 60.64) than soil samples (229 ± 64.17) measured by the observed number of ASVs. Likewise, fungal community diversity, measured by Shannon index and Faith’s PD, decreased in the rhizosphere. Shannon diversity index for fungi in rhizosphere samples ranged from 1.19 to 4.27 (average 2.87 ± 0.85), while in soil samples, this index ranged from 2.11 to 4.31 (average 3.43 ± 0.59). Faith’s PD ranged from 11.59 to 48.94 (average 30.82 ± 10.49) in rhizosphere samples, and in soil samples, this index ranged from 21.74 to 61.62 (average 42.20 ± 8.94) (**Figure 5A**). Regarding bacterial community diversity, it was observed that rhizosphere samples have lower bacterial diversity than soil samples (**Figure 5B**). For the Shannon diversity index, the value of rhizosphere samples (5.88 ± 0.77) was lower than that of soil samples (6.72 ± 0.42). Likewise, Faith’s PD was lower in rhizosphere samples (171 ± 42.23) than in soil samples (214 ± 35.26). Additionally, rhizosphere samples (171 ± 72) have lower bacterial species richness than soil samples (1,581 ± 339). Statistical comparison between low and high *Meloidogyne* densities in soil and rhizosphere samples showed no difference in any of the alpha diversity indices studied (**Supplementary Figure 7**).

**Figure 6:**
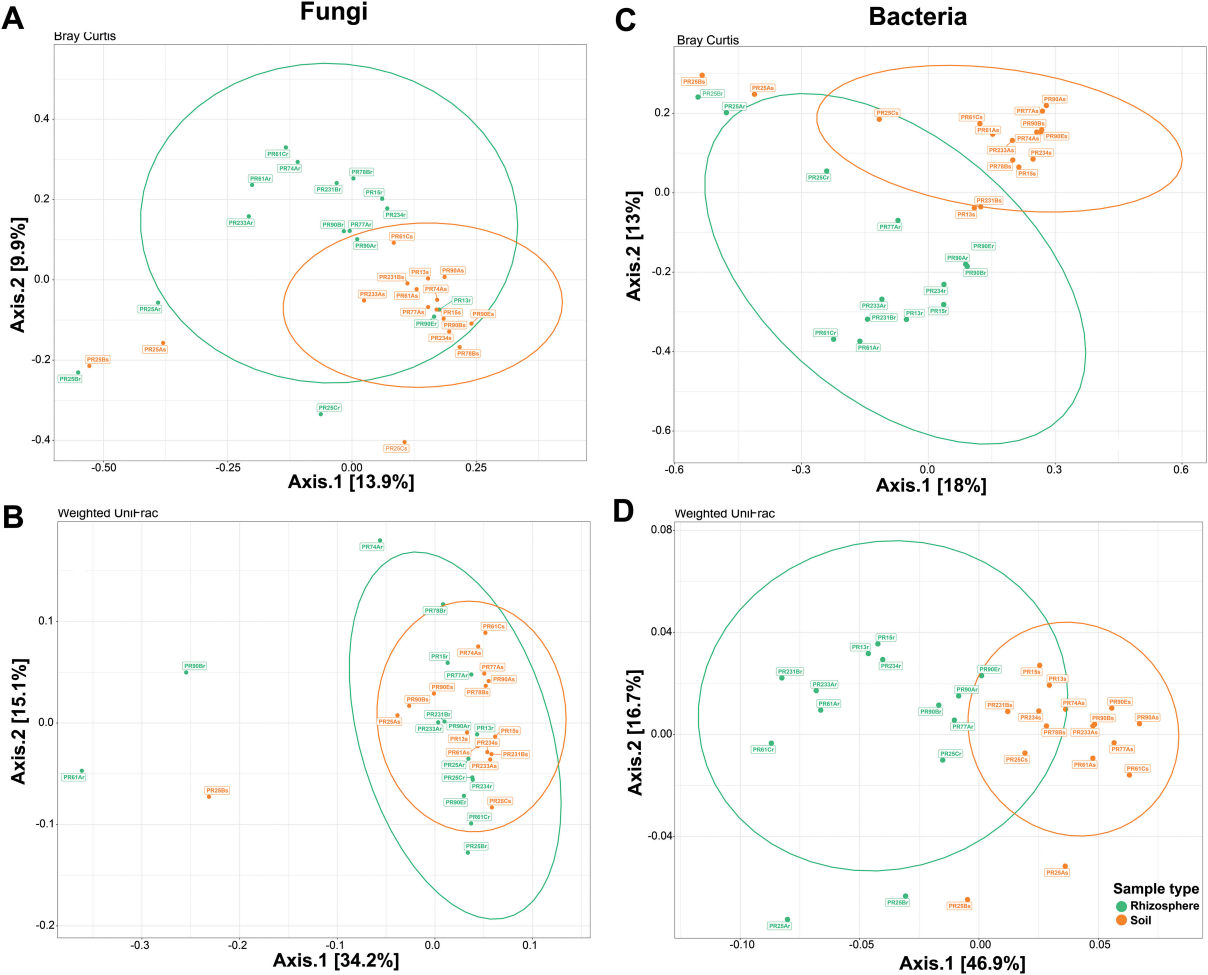
Beta diversity ordinations of the fungal and bacterial communities. Principal coordinate analysis (PCoA) ordinations of the fungal community based on Bray–Curtis dissimilarity **(A)** and weighted UniFrac distance **(B)** between soil (orange) and rhizosphere samples (green). PCoA ordinations of the bacterial community based on Bray–Curtis dissimilarity **(C)** and weighted UniFrac distance **(D)** between soil and rhizosphere samples. Bray–Curtis dissimilarity beta diversity measure for fungal communities was statistically significant (*p* = 0.003 by PERMANOVA), whereas weighted UniFrac distance beta diversity measure for fungal communities was not statistically significant (*p* = 0.233 by PERMANOVA). Both beta diversity measures for bacterial communities were statistically significant (*p* = 0.001 by PERMANOVA).

The Bray–Curtis and weighted Unifrac distances between rhizosphere and soil samples were visualized with a principal coordinate analysis (PCoA) (**Figure 6**). Bray–Curtis based on PCoA showed that fungal communities were grouped according to the type of sample (**Figure 6A**). However, the phylogenetic diversity of the rhizosphere fungal community was not statistically different from that of the soil fungal community. UniFrac-based PCoA showed that the rhizosphere and soil fungal communities overlapped (**Figure 6B**). Considering weighted UniFrac distance and Bray–Curtis dissimilarity, PCoA analyses showed a separate cluster between rhizosphere bacterial community and soil bacterial community (**Figures 6C, D**).

**Figure 7:**
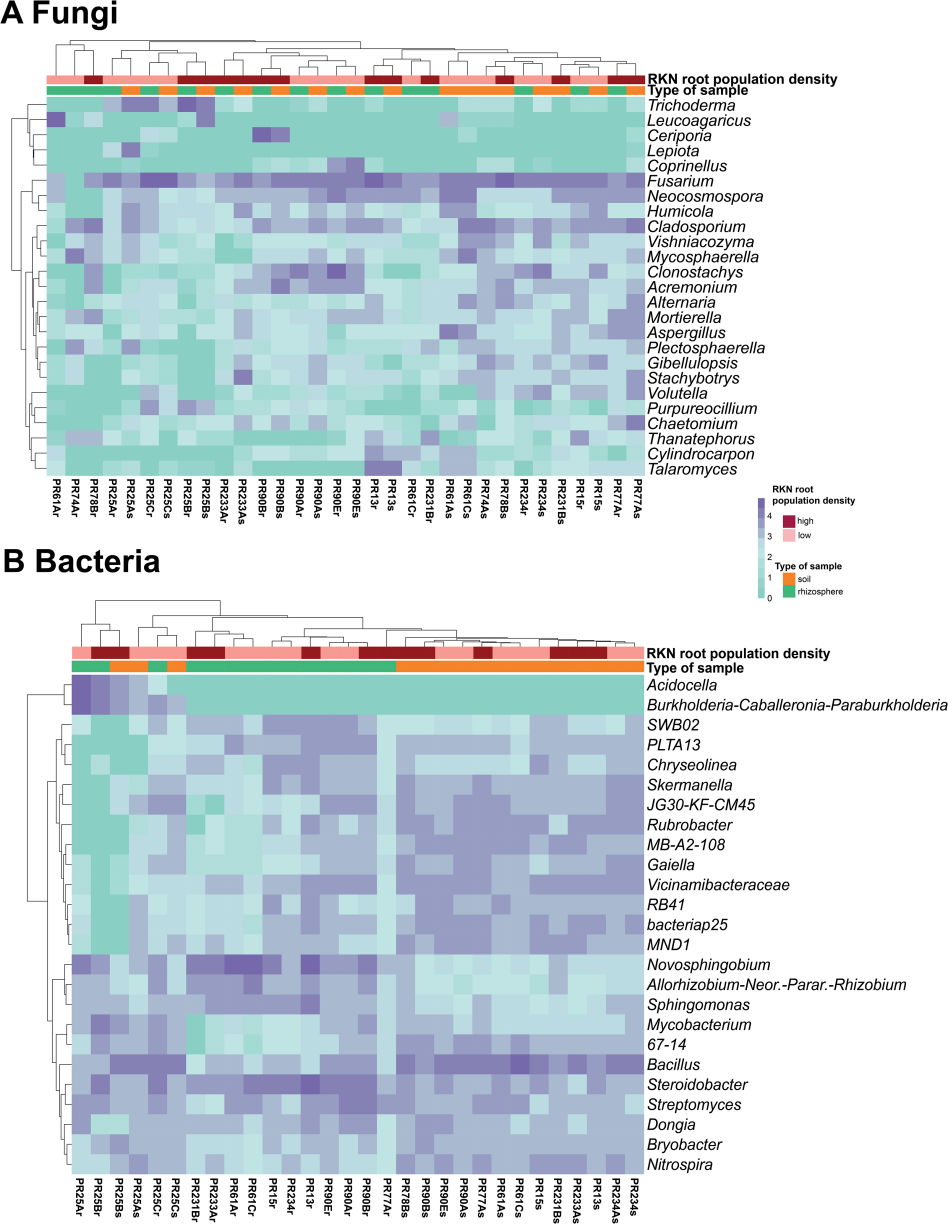
Heatmap of relative abundance (log_10_ transformed) of the 25 most abundant taxa at the genus level of fungal **(A)** and bacterial **(B)** microbiota composition. The horizontal clustering indicates the similarity of genus richness in different samples. The color of heatmap displays the relative abundance of each taxon across all samples.

The top 25 fungal and bacterial genera with the highest relative abundance were shown in the heatmaps (**Figure 7**) to identify similarity and differences between microbial communities according to sample type and *Meloidogyne* densities. With respect to fungi, *Fusarium*, *Cladosporium*, and *Neocosmospora* were the most relatively abundant genera. Rhizosphere fungal communities were not observed to form two different clusters between rhizosphere and soil samples (**Figure 7A**). Regarding bacteria, *Bacillus* and *Steroidobacter* were the most relatively abundant bacterial genera. Hierarchical clustering showed that rhizosphere samples tended to cluster together, separating the rhizosphere bacterial community from the soil community, with the exception of Carmona samples (field PR25) (**Figure 7B**). Some bacterial genera, such as *Novosphingobium*, *Allorhizobium–Neorhizobium–Pararhizobium–Rhizobium*, and *Sphingomonas*, showed a higher relative abundance in rhizosphere samples than in soil samples, whereas other bacterial genera had a lower relative abundance in rhizosphere samples than in soil samples, including *Skermanella*, *JG30-KF-CM45*, *Rubrobacter*, *MB-A2-108*, *Gaiella*, *Vicinamibacteraceae*, *RB41*, *bacteriap25*, and *MND1*. The genera *Acidocella* and *Burkholderia*–*Caballeronia*–*Paraburkholderia* are clearly associated with acidic soil in Carmona (field PR25).

To identify fungal and bacterial taxa associated with *Meloidogyne* densities, LEfSe analyses were conducted. Different fungal and bacterial taxa were detected characterizing samples with low and high *Meloidogyne* density depending on the type of sample, rhizosphere, or soil. In the rhizosphere, no fungal taxon was selected as differentially abundant between samples with low and high *Meloidogyne* densities. In soil samples, two fungal genera (*Mycosphaerella* and *Sporobolomyces*) and one class (Sordariomycetes) were enriched in samples with low *Meloidogyne* densities, whereas one genus (*Cephaliophora*) and one order (Tremellales) were significantly more abundant in samples with high *Meloidogyne* densities (**Supplementary Figure 8A**). The rhizosphere of samples with low *Meloidogyne* densities was characterized by the predominance of five bacterial genera (*Arthrobacter*, *PAUC26f*, *Planctomycetales*, *Tagaea*, and *Virgisporangium*) and one family (Xanthomonadaceae), while three bacterial genera (*Aestuariicella*, *Cellvibrio*, and *Prosthecobacter*) were enriched in the rhizosphere of samples with high *Meloidogyne* density (**Supplementary Figure 8B**). Finally, only two bacterial genera (*Clostridium sensu stricto 8* and *Nitrososphaeraceae*) were identified as predominant in soil samples with low *Meloidogyne* density, whereas the soil of samples with high *Meloidogyne* density was characterized by the abundance of seven bacterial genera (*Dyadobacter*, *Fluviicola*, *Niabella*, *Permianibacter*, *Polyangium brachysporum group*, *Promicromonospora*, and *Pseudogulbenkiania*) (**Supplementary Figure 8C**).

The nematophagous fungi detected by real-time qPCR and isolated from *Meloidogyne* eggs (**Table 3**) were compared with those identified by high-throughput sequencing (**Supplementary Figure 9**). *Arthrobotrys dactyloides*, *A. oligospora*, *Catenaria* sp., and *P. lilacinum* were detected in the same samples by both real-time qPCR and metabarcoding. *Purpureocillium lilacinum* and *Pochonia chlamydosporia* were isolated from *Meloidogyne* eggs in one and three almond groves, respectively, although both species were detected in all almond groves by metabarcoding, such as *Trichoderma asperellum*, which was identified parasitizing *Meloidogyne* eggs in one almond grove, but it was detected in three groves by metabarcoding. It should be noted that *T. asperellum* was isolated from *Meloidogyne* eggs in the almond grove where a higher abundance of reads assigned to this species was detected, while the other two almond groves where this species was detected by metabarcoding showed a lower abundance of reads (**Supplementary Figure 9**).

### 3.7 Functional analyses

**Figure 8:**
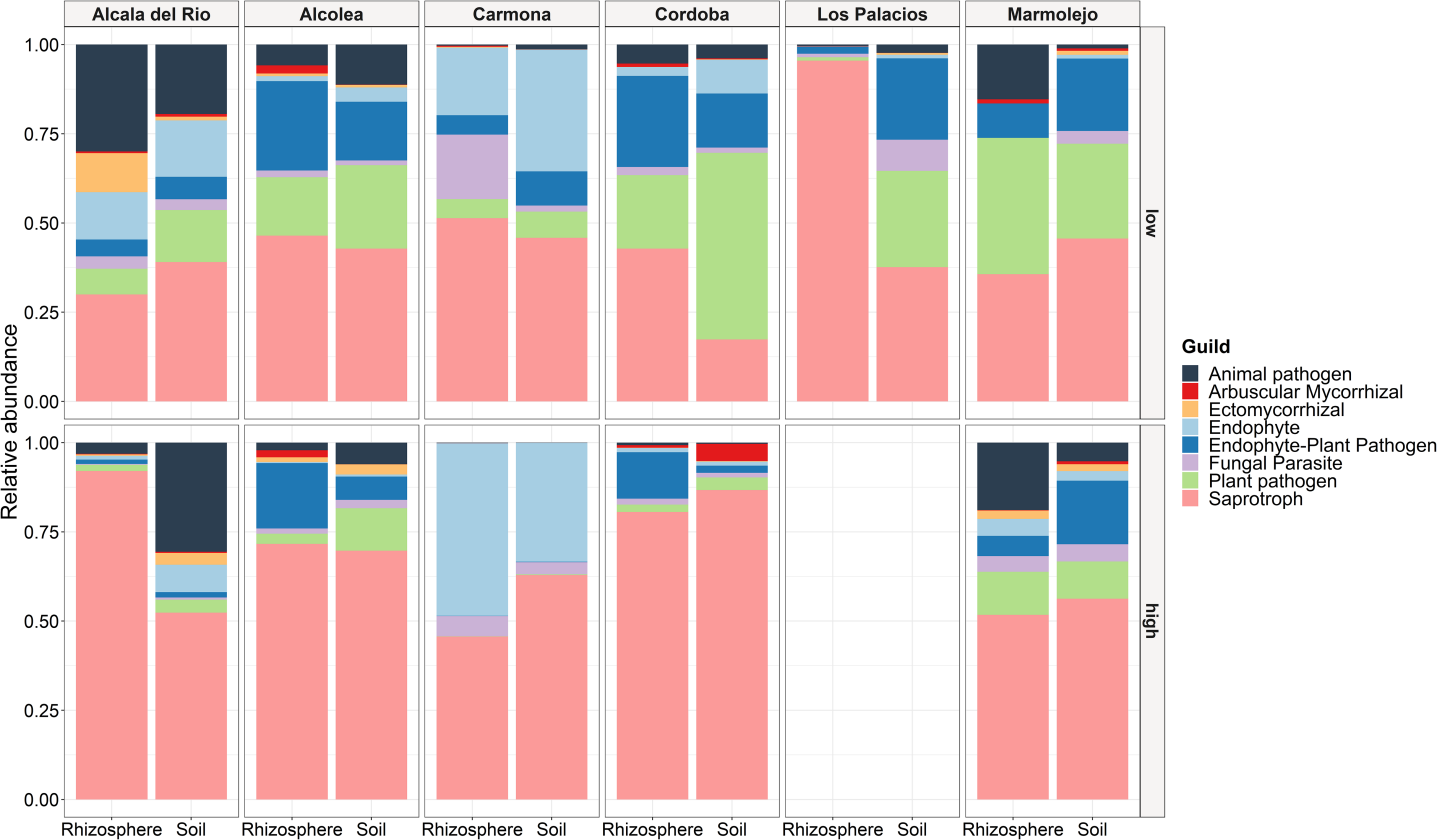
Relative abundance of fungal functional guilds between different localities, type of sample (soil and rhizosphere), and *Meloidogyne* population density in roots [low (<600 eggs per g of root) and high (>600 eggs per g of root)].

A total of 830 fungal ASVs (69.51% of the total filtered ASVs) were assigned to any guild and trophic mode. After excluding assignments with a confidence of “possible,” 565 ASVs (47.32% of the total filtered ASVs) were considered. These ASVs were assigned to seven trophic modes and 42 guilds, which were merged into eight fungal functional guilds: animal pathogen, arbuscular mycorrhiza, ectomycorrhiza, endophyte, endophyte-plant pathogen, fungal parasite, plant pathogen, and saprotroph. Among the eight guilds defined here, most fungi were classified into the saprotroph guild, indicating a higher ecosystem function related to decomposition (**Figure 8**). Saprotrophs were the most abundant guild with a relative abundance from 17.37% to 95.49% (average 55.46%), followed by endophytes (relative abundance from 0% to 48.35%, average 13.87%), plant pathogens (relative abundance from 0.09% to 52.31%, average 10.63%), endophyte-plant pathogens (relative abundance from 0.07% to 25.52%, average 8.27%), and animal pathogens (relative abundance from 0.09% to 30.58%, average 14.51%), while fungal parasites, ectomycorrhizae, and arbuscular mycorrhizae were found in low frequency, with relative abundances of 0%–18.09% (average 3.97%), 0%–10.94% (average 1.07%), and 0%–4.82% (average 0.48%), respectively. Saprotrophs showed an increasing trend with *Meloidogyne* density. The relative abundance of saprotrophs was higher in samples with high *Meloidogyne* densities, whereas plant pathogens and endophyte-plant pathogens showed higher relative abundance in samples with low *Meloidogyne* density (**Figure 8**).

Functional gene annotations predicted by PICRUSt2 identified a total of six primary pathways and 41 secondary pathways. The relative abundance of KEGG primary pathways are shown in **Supplementary Figure 10**, which illustrates that the predicted functions at all samples were dominated by metabolism-related pathways. Specifically, metabolism-related pathways exhibited a relative abundance from 68.53% to 71.08% (average 70.01%). In particular, within the primary metabolism-related pathway, carbohydrates and amino acid metabolism pathways were the dominant secondary pathways. The second and third most abundant primary pathways were those related to genetic information processing (relative abundance from 11.93% to 14.76%, average 13.43%) and environmental information processing (relative abundance from 8.79% to 10.77%, average 9.82%), while pathways related to cellular processes (relative abundance from 3.45% to 4.63%, average 3.87%), human diseases (relative abundance from 1.30% to 2.25%, average 1.69%), and organismal systems (relative abundance from 1.12% to 1.27%, average 1.18%) accounted less than 10% of relative abundance. No trend was observed in the relative abundance of the bacterial functional predictions with respect to *Meloidogyne* density levels (low–high). The differential abundance analysis performed with the *ggpicrust2* package revealed no statistically significant differences (p > 0.05) in KEGG pathways between low and high *Meloidogyne* densities.

## 4 Discussion

Control methods and management strategies against RKNs include prevention measures, methods to reduce nematode populations, such as crop rotation, solarization, nematicides, resistant/tolerant rootstocks, and biological control. Knowledge of soil and rhizosphere microbiota associated with almond crops, as well as their interactions with *Meloidogyne* spp., is essential to stablish new sustainable pest management strategies. Only a few researches are trying to understand the relationships of soil and root microorganisms (bacterial and fungi) with *Meloidogyne* plant infection from different point of views, such as temporal (Yergaliyev et al., 2020), nematode density (Zhou et al., 2019; Lu et al., 2023), and microbiomes in the root, soil, and nematode (Cao et al., 2015; Tian et al., 2015; Toju and Tanaka, 2019; Lamelas et al., 2020; Masson et al., 2020; Yergaliyev et al., 2020). Yergaliyev et al. (2020) found that bacterial gall communities differed from root segments lacking the gall in eggplant, and this structure is maintained throughout the crop season. These bacteria were modulated in connection with root structure modifications, polysaccharide metabolism, and chitin metabolism (Yergaliyev et al., 2020). Bacteria in the gall are often found in hypotoxic and anaerobic environments, and this could lead to infective juveniles to select uninfected root regions (Yergaliyev et al., 2020). The diversity of the rhizospheric bacteria gradually changed with the increasing severity of RKN infection in tobacco plants (Lu et al., 2023). However, all of these studies are based on herbaceous plants (many of them using tomato). This research provides some new insights into the *Meloidogyne* and plant relationships in mature deciduous woody trees (almond grafted into the hybrid rootstock GF-677) with different levels of *Meloidogyne* density in roots and their interaction with soil microbiota, which has not been explored to our knowledge. Deciduous woody trees have some different ecological traits vs horticultural crops, such as negligent root growth during a period of time (winter) and pushes of root growth during spring and summer. Additionally, rootstocks are classified by vigor, and the main susceptible hybrid rootstock used in Spain (GF-677) is classified as vigorous. The holistic point of this paper (soil nematode, fungal and bacterial soil and rhizosphere densities, bacterial and fungi diversity, and percentage of different taxa using metabarcoding, specific real-time qPCR to detect and quantify biocontrol agents in soil nematodes and parasitism levels in eggs of our samples) gives data and cues about the processes occurring in the rhizosphere and soil niches among different microorganisms.

Our selected almond groves showed soil degradation in the majority of sampling points based on the data obtained analyzing the nematode communities. Nematodes represent several trophic groups, fulfill important roles in ecosystem processes, and respond rapidly to environmental disturbance with good ecological indices for their analysis (Neher, 2001; Du Preez et al., 2022). In this sense, the densities of predatory nematodes were not related with the levels of *Meloidogyne* in soil or in the roots. This could be related to more susceptibility to these K-strategist nematodes with a longer generation time of months, producing few but large eggs, and they cannot rapidly respond numerically to new food resources (Bongers and Bongers, 1998). In this case, the soil movement in the grove to create flattening and grooves and the intensification of the almond crop with irrigation and high amounts of fertilizers and chemicals through drip irrigation for several years (during the tree life) could change the soil ecosystem for these sensitive microorganisms and other microbes in the soil (i.e., BCAs). Additionally, the biocontrol effects in perennial crops must be maintained during long periods of time, and in some cases, these crops have deeper root distribution than annuals, which means that BCAs need to operate deeper complicating the action of some biocontrol agents (Stirling, 2014). In this sense, the levels of fungal parasites of *Meloidogyne* eggs were found in a good prevalence (56.25% of the samples), but in a low incidence parasitizing eggs in the roots (from 1% to 8%). This low level of egg infections has been related with the size of the gall and the root infection levels, in which bigger galls creates a more protected environment for eggs in the egg mass than in smaller galls with more exposed egg masses to the soil (Kerry, 2000), or the depth at which the *Prunus* rootstock roots are found maybe are not coupled with these biocontrol agent’s requirements. Additionally, in all groves, the drip irrigation is the way to irrigate and fertilize plants and where high concentrations of chemicals could be found, which could affect the establishment of these biocontrol agents. In some fewer intensive crops in the area of study, such as olive, the irrigation increased the abundance (not the presence) of *P. lilacinum* and *Hirsutella rhossiliensis* (Campos-Herrera et al., 2022). Stirling et al. (1979) found that *Dactylella oviparasitica* controlled the infection of *Meloidogyne* spp. in the rootstock Lovell in California. In our sampling, the fungal egg parasitism was conducted by *P. chlamydosporia*, *P. lilacinum*, and *Trichoderma asperellum*. The most frequent fungal species isolated was *P. chlamydosporia*, which was found in three of the six (50%) commercial almond groves. Surprisingly, even with the prevalence of *P. lilacinum* in all sampled fields and at different *Meloidogyne* levels detected by real-time qPCR in the extracted soil nematodes, the controlling effect in *Prunus* rootstock-embedded *Meloidogyne* egg masses was low. *Pupureocillium lilacinum* has been effective using 7.5 × 10^3^ or 1 × 10^4^ chlamydospores gram of soil in pot experiments from a strain isolated in peach against an inoculum of second-stage juveniles of *M. javanica* (Saeed et al., 2023). We could suggest that *P. lilacinum* did not colonize the *Prunus* trees, or other consortia of microorganisms reduce the ability to infect egg masses and nematodes. As mentioned before, the position of the egg masses in woody plants could differ and reduce the penetration efficacy. Ratios between fungi and bacteria are also showing a bacterial-dominated environment in *Prunus* groves managed conventionally, with the exception of Carmona grove, which has a very low pH and high levels of sand, which could induce the presence of a more dominating fungi environment.

According to the nematode community analysis, most of the soils sampled were classified as disturbed or degraded soils. Another measure that can be used as indicator of soil status is the fungal:bacterial ratio. The low fungal:bacterial ratio of our samples (ranging from 0.0034 to 0.092) would also indicate the low soil quality of the sampled almond groves. Fungal-dominated ecosystems can sequester more carbon (C) than bacterial-dominated communities and have been associated with a qualitative and quantitative enhancement of organic matter and greater self-regulation (Bardgett and McAlister, 1999; Six et al., 2006). Therefore, these results and those of a previous study (Clavero-Camacho et al., 2024) showed that the intensification of the almond crop in recent years could increase the damage of PPN and reduce the soil quality. To improve the soil quality of almond groves in Spain, practices, such as reduced tillage or no-tillage, reduction of agrochemicals, introduction of organic farming practices, or cover crops, are needed to increase the microbial biomass toward a fungal-dominated community and thus increase C sequestration (Six et al., 2006).

The molecular tools used in this study, including real-time qPCR and metabarcoding by high-throughput sequencing, allowed the identification of nematophagous fungi in the rhizosphere and soil of GF-677 almond rootstocks, and with both techniques, we obtained identical results. Some of these fungi were isolated from *Meloidogyne* eggs, thus confirming that these can act as parasites of root-knot nematodes. The presence in rhizosphere and soil samples of fungi isolated by spreading *Meloidogyne* eggs on a growth-restricting medium was confirmed by molecular tools. However, some nematophagous fungi were identified in some samples by real-time qPCR and metabarcoding, but these were not isolated from *Meloidogyne* eggs. This could be due to the extraction process of *Meloidogyne* eggs, as the egg masses cannot be handpicked because of the hardness of the woody roots of *Prunus*. On the other hand, fungi, such as *P. chlamydosporia* and *P. lilacinus*, are saprophytes, so these can grow on a wide range of organic materials (Stirling, 2014). Therefore, the study of the parasitism of *Meloidogyne* eggs may be useful to confirm that these fungi act as nematophagous fungi.

There are important diversity and composition differences between the rhizoplane and the soil microbiota in the sampled groves. The lower diversity found in the rhizoplane could be associated to a selection of microorganism by the root and the bigger soil amount included in the soil sample (approximately 0.5 g). Additionally, the majority of the ASVs are shared between the rhizoplane and the soil suggesting that additionally, through the selection by the plant, many of the microbial species came from the nearby soil (Berg and Smalla, 2009). The fungal phyla Ascomycota and Basidiomycota and bacterial phyla Proteobacteria, Actinobacteriota, Acidobacteriota, and Bacteroidota were dominant in both soil and rhizosphere. These results are in agreement with those reported in a previous study on the soil microbial community of almond crops under different management strategies (Camacho-Sanchez et al., 2023). The fungal orders Hypocreales, Capnodiales and Sordariales, and fungal families Nectriaceae, Cladosporiaceae, and Chaetomiaceae were also dominant in this previous study (Camacho-Sanchez et al., 2023), while the dominant bacterial orders and families found in our study differ from those reported by Camacho-Sanchez et al. (2023). In our research, the bacterial orders Rhizobiales, Burkholderiales, Sphingomonadales, and Bacillales, and bacterial families Sphingomonadaceae, Bacillaceae, and Gemmatimonadaceae stood out, whereas the orders Vicinamibacterales, Gemmatimonadales, and Chitinophagales, and the families Gemmatimonadaceae, Vicinamibacteraceae, and Chitinophagaceae were reported as dominant by Camacho-Sanchez et al. (2023). Our sampling is geographically wider than that of Camacho-Sanchez et al. (2023), which could also explain the difference patterns in the levels and prevalence of some microbial interacting with *Prunus* roots. Our results showed a significant variation in fungal and bacterial diversity, both alpha and beta diversity, that could be attributed to the type of sample (rhizosphere or soil). The geographical location of the samples probably affects the diversity and composition of fungal and bacterial communities more than the *Meloidogyne* density. PCoA analyses showed that samples of the same almond groves tended to cluster together. In particular, Carmona samples (PR25) appear separated from the rest of the samples, which could be due to the different soil psychochemical properties. In addition, these samples showed differences in community composition, specifically, some bacterial genera were more abundant (*Acidocella* and *Burkholderia–Caballeronia–Paraburkholderia*) and associated with acidic soils (Kishimoto et al., 1995; Hetz and Horn, 2021), while other genera presented a low abundance (*SWB02*, *PLTA13*, *Chryseolinea*, *Skermanella*, *JG30-KF-CM45*, *Rubrobacter*, *MB-A2-108*, *Gaiella*, *Vicinamibacteraceae*, *RB41*, *bacteriap25*, and *MND1*) in these samples. Additionally, for the vigorous hybrid rootstock GF-677 that could tolerate better the *Meloidogyne* parasitism than other horticultural crops, such as tomato, our sampling is based on natural infected roots in which different infection timings could dilute the response to the plant and, in this sense, affect the plant response, which could conduct different exudates and relationships with the soil microbiota. However, several fungal and bacterial taxa, and different fungal functions could be found as differential in low and high levels of *Meloidogyne* eggs in the root. The increase in saprotrophic function in fungal community in soil and rhizosphere at high levels of nematodes infecting the roots (with the exception of rhizosphere in acidic soils in Carmona samples and similar levels of this function in the soils of Marmolejo samples) indicates that the plant is increasing the levels of nutrients in the roots, and the nematode feeding sites are creating a sink for photoassimilates. Carbon partitioning analysis showed that nematodes are strong metabolic sinks and significantly change the carbon distribution pattern in soybean *Meloidogyne*-infected plants (Carneiro et al., 1999). However, this differential response of different functions is not retained for bacteria in low and high levels of *Meloidogyne* in the roots. Only a few fungi and bacterial differential taxonomic groups were found associated with different levels of *Meloidogyne* in the rhizosphere and the soil. The highly different microbiota found between the sampled groves (**Figure 6**) could make this analysis difficult to find differential genera between high and low levels of *Meloidogyne*. However, this specific data about taxa differentiation is difficult to explain because we could not find a clear trend in the large diversity of functions found in these fungi and bacteria taxonomic groups. Specifically, ecological constraints or competence among other species in the ecosystem for each species could influence in their selection.

In conclusion, this work explores the effects of soil microbiota, nematodes, and BCAs in several almond fields infected with different gradient of *Meloidogyne* infecting almond roots. The data showed the scarce impact of BCAs and predator nematodes in conventional almond production to the regulation of *Meloidogyne* in the field, even with their presence in the field. The data also showed that *Meloidogyne* infection does not change dramatically the root microbiome in adult trees, probably because of the vigorous growth of the susceptible hybrid rootstock GF-677. However, fungal saprotrophism function in the microbiome is altered by the infection of the nematode at high levels in rhizosphere and soil. This study reveals the soil complexity in field experiments and the need for a better understanding of the underground relationship between plant–fungi–bacteria–nematodes to choose better practices than promote biocontrol of *Meloidogyne* in the field.

## Data Availability

The datasets presented in this study can be found in online repositories. The names of the repository/repositories and accession number(s) can be found below: https://www.ncbi.nlm.nih.gov/, Bioproject: PRJNA993485; https://www.ncbi.nlm.nih.gov/, OR801652-OR801667.
