# Supplementary material for: Diversity of microbial, biocontrol agents and nematode abundance on a susceptible Prunus rootstock under a Meloidogyne root gradient infection
Source: Front Plant Sci. 2024 Sep 23;15:1386535. doi: 10.3389/fpls.2024.1386535 (PMC11456498; doi:10.3389/fpls.2024.1386535)
Supplement: Supplementary file 1 [file DataSheet1.pdf]

Supplementary Material

Diversity of microbial, biocontrol agents and nematode abundance on a susceptible *Prunus* rootstock under a *Meloidogyne* root gradient infection

Ilenia Clavero-Camacho[1,2], Alba N. Ruiz-Cuenca[1,3], Carolina Cantalapiedra-Navarrete[1], Pablo Castillo[1], Juan E. Palomares-Rius[1,*]

*** Correspondence:** Juan E. Palomares-Rius: palomaresje@ias.csic.es

1.1 Supplementary Figures

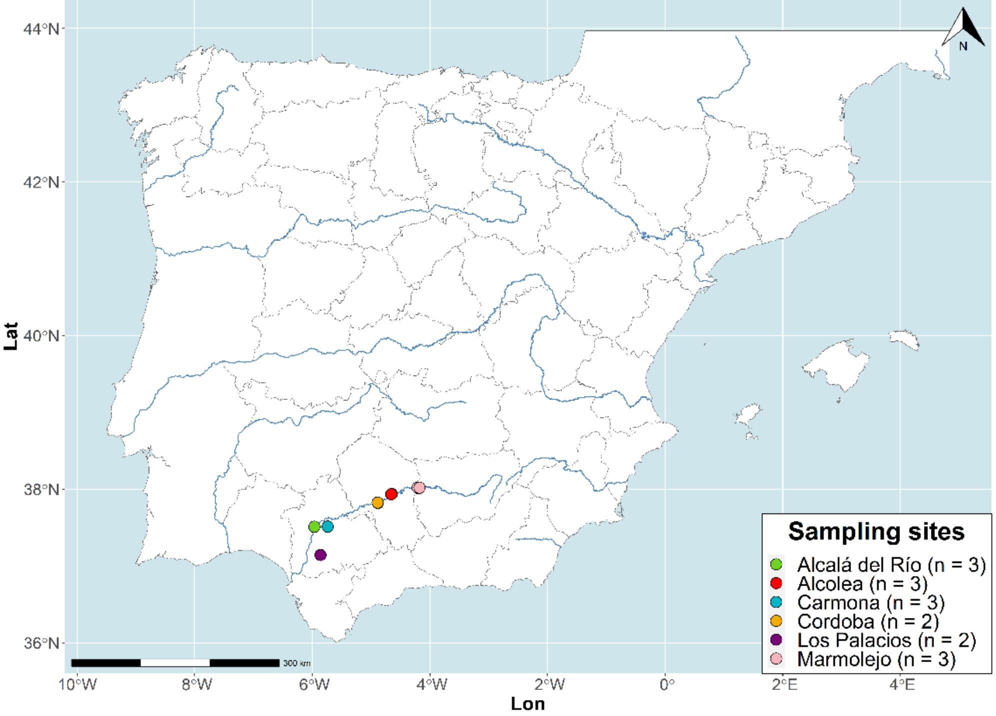

Supplementary Figure 1. Geographical locations of the 16 sampling points classified into six localities according to commercial almond groves.

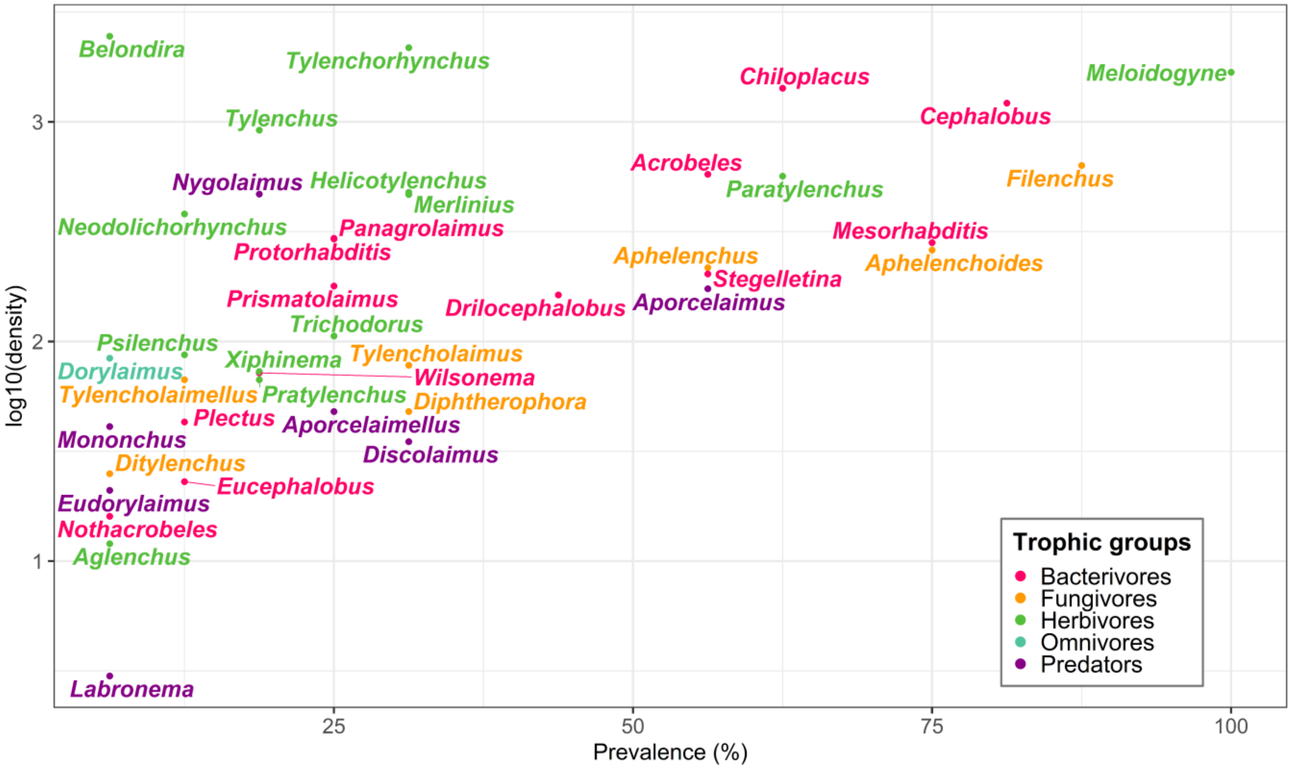

Supplementary Figure 2. Dominance diagram of nematode genera identified in the rhizospheric soil of almond (*Prunus dulcis* var. *dulcis*). Density: mean of nematodes of each genus per 500 cm^3 of soil in all sampling points where that genus was detected. Prevalence: percentage of samples in which a nematode genus was detected with respect to total number of samples processed.

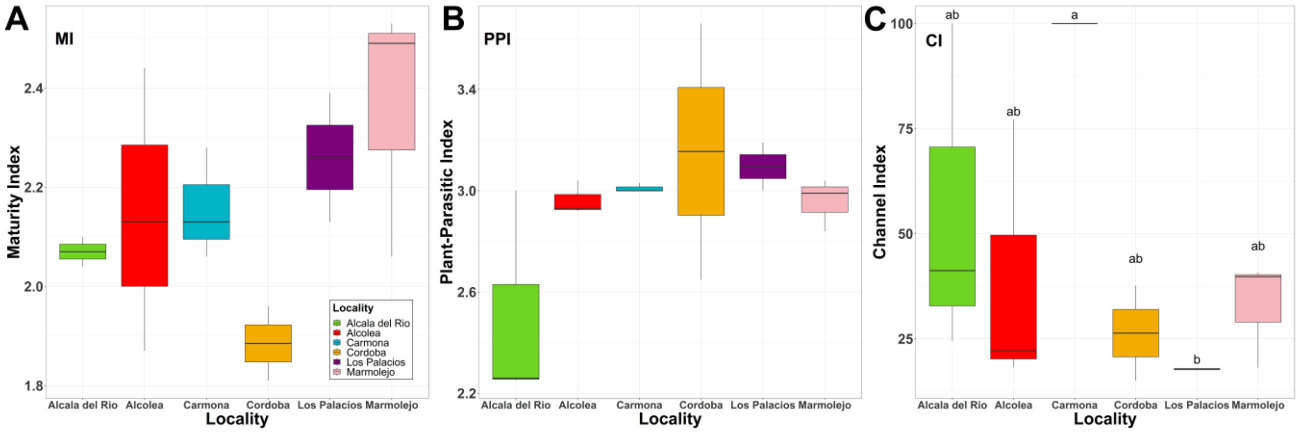

Supplementary Figure 3. Effects of geographic location of the six commercial almond groves studied on ecological and functional indices of soil nematodes. Maturity index for free-living nematodes (MI) (A). Plant-parasitic index (PPI) (B). Channel index (CI) (C). All indices were calculated with NINJA software and visualized and analysed with R software. Only statistically significant differences were found for the variable CI. Different letters indicate significant differences between localities according to HSD test ($P < 0.05$).

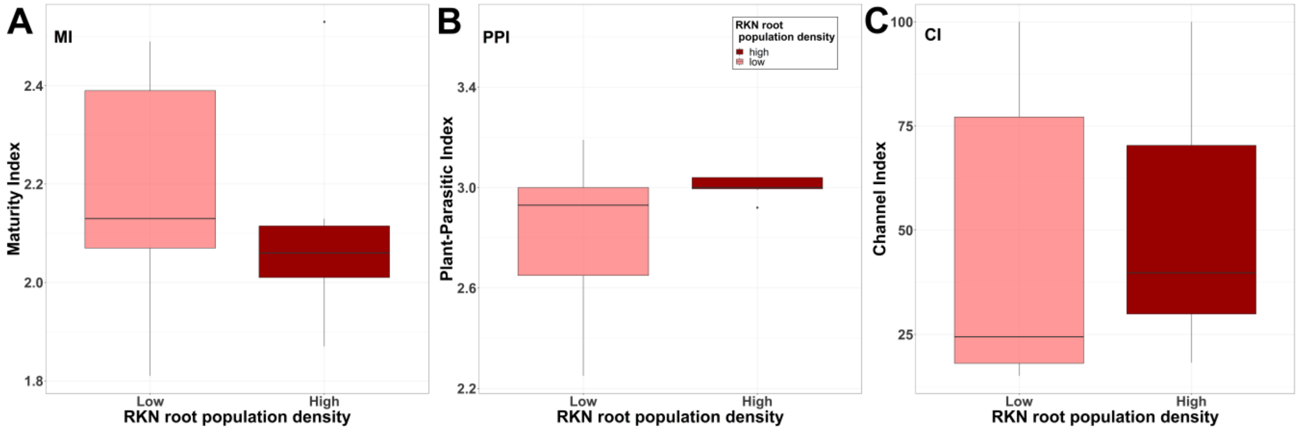

Supplementary Figure 4. Effects of *Meloidogyne* population density in roots (low and high) on ecological and functional indices of soil nematodes. Maturity index for free-living nematodes (MI) (A). Plant-parasitic index (PPI) (B). Channel index (CI) (C). All indices were calculated with NINJA software and visualized and analysed with R software. The statistical analyses found no significant differences for any variable ($P > 0.05$).

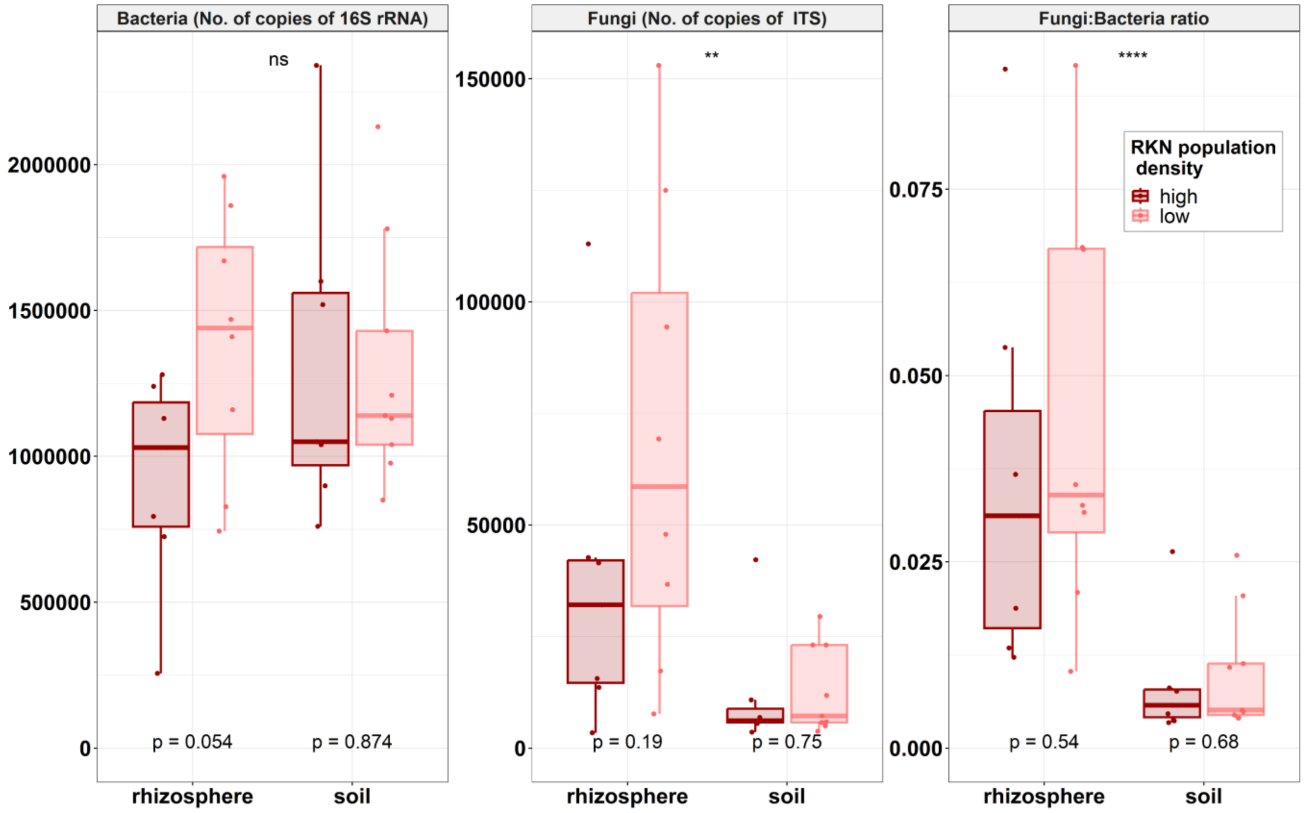

Supplementary Figure 5. Fungal and bacterial abundances in soil and rhizosphere samples based on real-time qPCR. Fungal:Bacterial ratio based on the number of copies of ITS region and 16S rRNA gene. Differences of Fungal:Bacterial ratio, ITS or 16S copy numbers between sample type (rhizosphere, soil) were evaluated (Wilcoxon, $P < 0.05$) and results are shown with the significance levels. While differences between *Meloidogyne* density (low, high) are shown with the formatted *P* value.

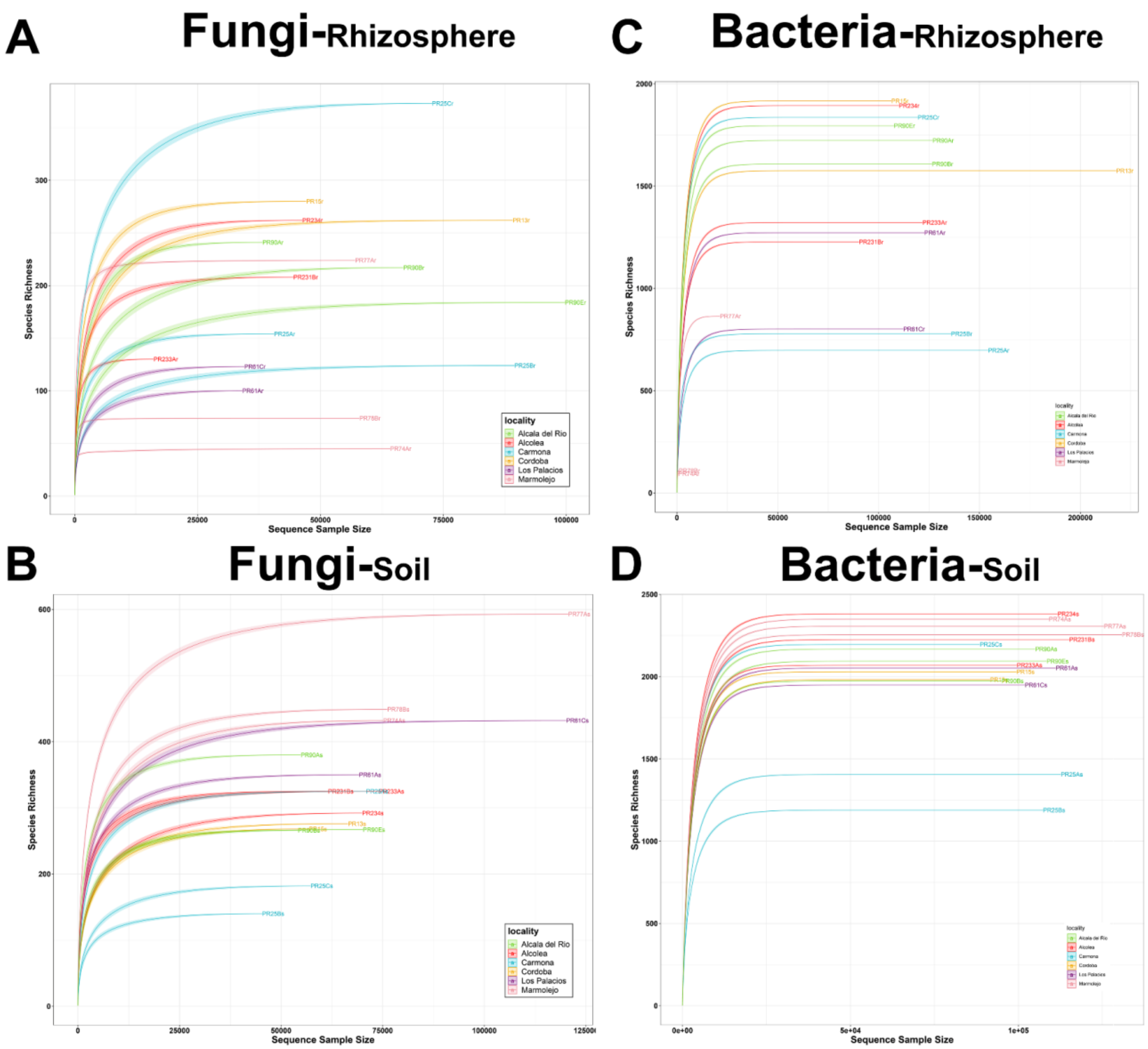

Supplementary Figure 6. Rarefaction curves showing the sequencing depth and observed ASVs. Rarefaction curves of communities of fungi (rhizosphere (A) and soil (B)) and bacteria (rhizosphere (C) and soil (D)).

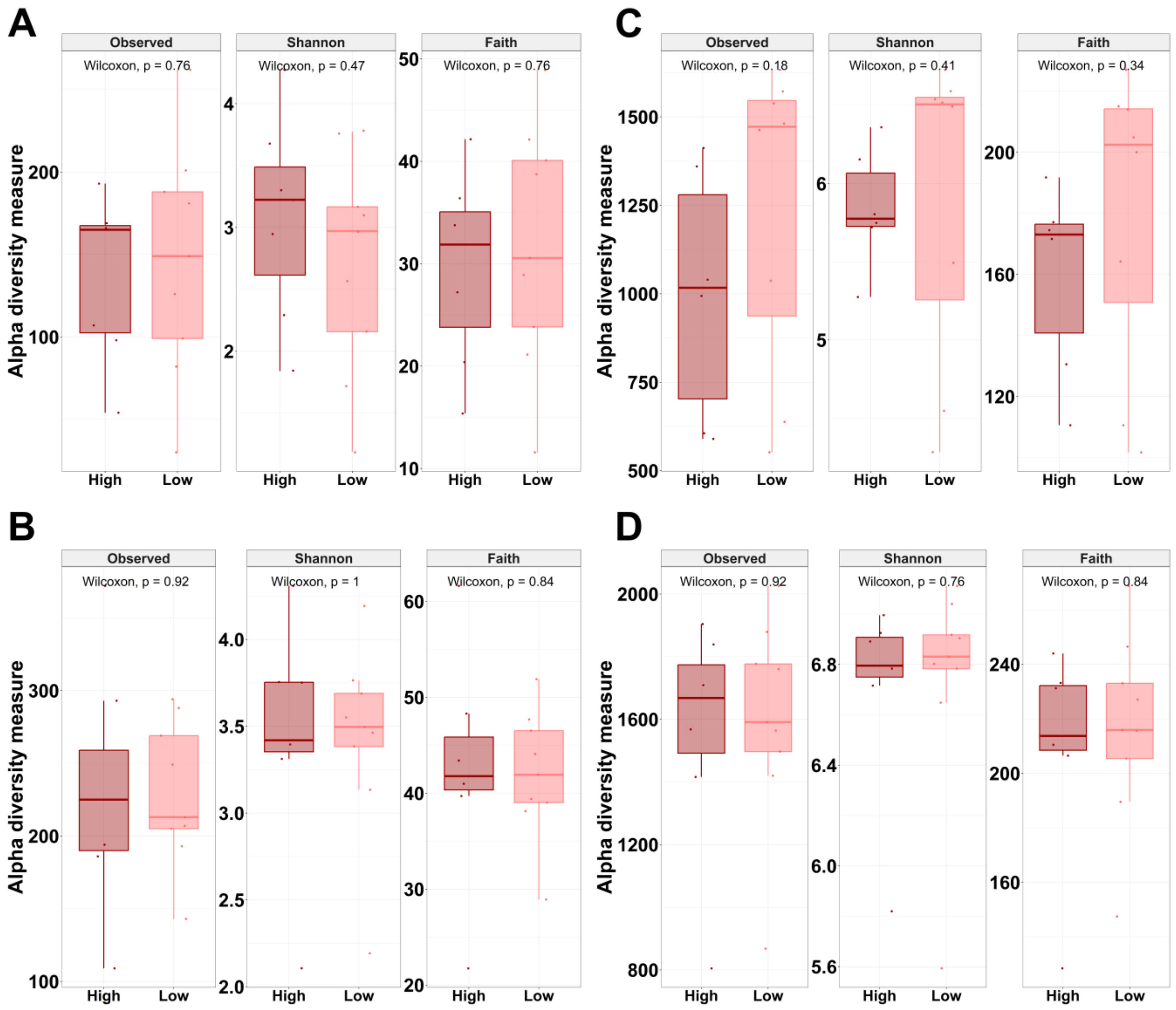

Supplementary Figure 7. Alpha diversity. Box plots illustrating the differences in the three metrics of alpha diversity (Observed ASVs, Shannon diversity index and Faith's phylogenetic diversity index) of the fungal communities between low and high *Meloidogyne* densities in rhizosphere (A) and soil (B) samples, and bacterial communities between low and high *Meloidogyne* densities in rhizosphere (C) and soil (D) samples. Comparisons were not statistically significant ($P > 0.05$).

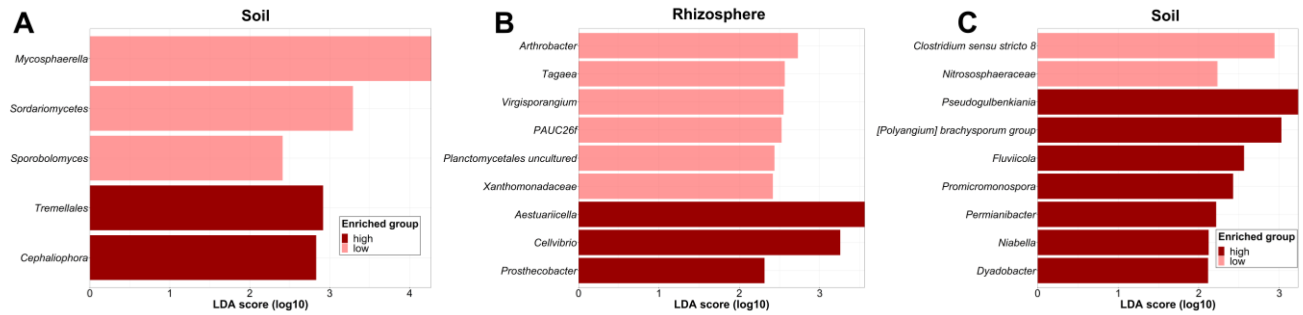

Supplementary Figure 8. LEfSe analyses to identify differences in abundant fungal and bacterial taxa, at genus level or above, between low and high *Meloidogyne* densities in soil (A, C) and rhizosphere (B) samples. Bar plots showing linear discriminant analysis (LDA) scores for fungi (A) and bacteria (B, C). Only taxa with a LDA significant threshold > 2 are shown. Fungal and bacterial genera enriched in samples with low *Meloidogyne* densities are shown in pink, while genera with higher abundance in samples with high *Meloidogyne* densities are indicated in red.

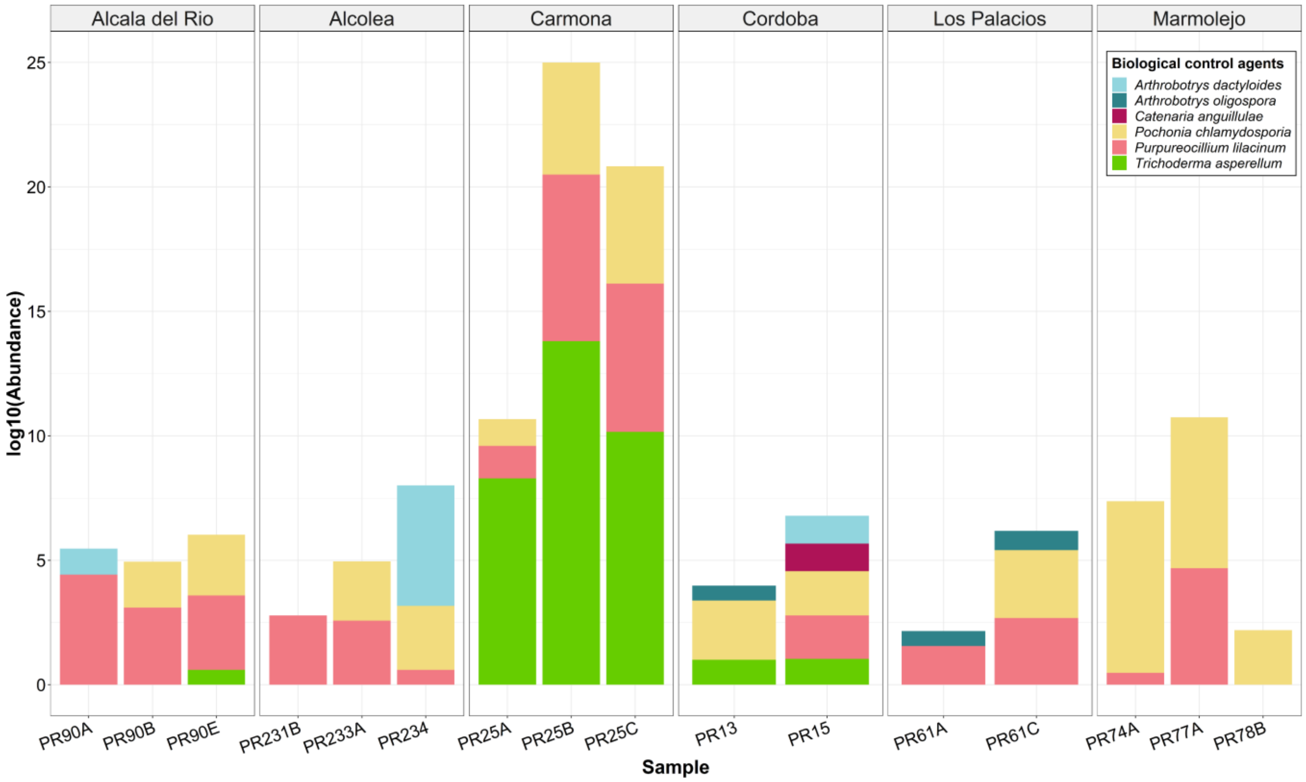

Supplementary Figure 9. Log$_{10}$ abundance of the six nematophagous fungi studied by real-time qPCR in this research, which were detected by high-trhoghput amplicon sequencing methods.

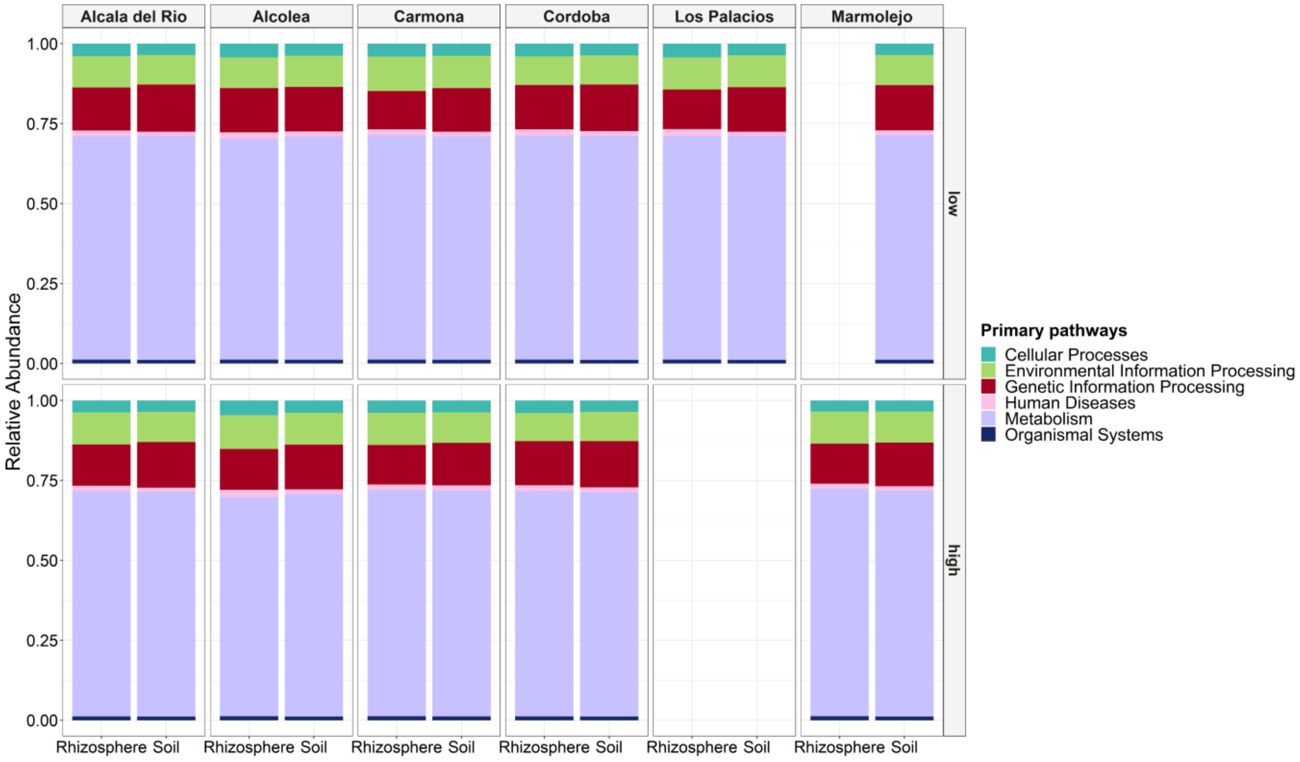

Supplementary Figure 10. Relative abundances of KEGG primary pathways between different localities, type of sample (soil and rhizosphere) and *Meloidogyne* population density in roots (low and high).

1.2 Supplementary Tables

Supplementary Table 1. Nematode population density (nematodes per 500 cm3 of soil, range and mean) and prevalence (%) of all nematode genera identified in the rhizosphere of almond (*Prunus dulcis* var. *dulcis*) in Andalusia (southern Spain).

Genus	Family	Trophic group	Total Soil Nematode	
			**Prevalence[a]**	**Density[b]**
Acrobeles	Cephalobidae	Bacterivores	56.25	577 (29-2232)
Cephalobus	Cephalobidae	Bacterivores	81.25	1217 (34-3926)
Chiloplacus	Cephalobidae	Bacterivores	62.50	1423 (32-10230)
Drilocephalobus	Osstellidae	Bacterivores	43.75	163 (25-375)
Eucephalobus	Cephalobidae	Bacterivores	12.50	23 (18-28)
Mesorhabditis	Rhabditidae	Bacterivores	75.00	282 (14-1428)
Nothacrobeles	Cephalobidae	Bacterivores	6.25	16 (16-16)
Panagrolaimus	Panagrolaimidae	Bacterivores	25.00	295 (53-1001)
Plectus	Plectidae	Bacterivores	12.50	43 (2-84)
Prismatolaimus	Prismatolaimidae	Bacterivores	25.00	179 (62-450)
Protorhabditis	Rhabditidae	Bacterivores	25.00	293 (79-471)
Stegelletina	Cephalobidae	Bacterivores	56.25	203 (41-572)
Wilsonema	Plectidae	Bacterivores	18.75	72 (16-159)
Aphelenchus	Aphelenchoididae	Fungivores	56.25	217 (10-586)
Aphelenchoides	Aphelenchoididae	Fungivores	75.00	261 (32-833)
Diphtherophora	Diphtherophoridae	Fungivores	31.25	48 (28-62)
Ditylenchus	Anguinidae	Fungivores	6.25	25 (25-25)
Filenchus	Tylenchidae	Fungivores	87.50	633 (21-4495)
Tylencholaimellus	Tylencholaimellidae	Fungivores	12.50	67 (7-127)
Tylencholaimus	Tylencholaimidae	Fungivores	31.25	78 (16-163)
Aglenchus	Tylenchidae	Herbivores	6.25	12 (12-12)
Belondira	Belondiridae	Herbivores	6.25	2455 (2455-2455)

Helicotylenchus	Hoplolaimidae	Herbivores	31.25	478<br>(32-1500)
Meloidogyne	Heteroderidae	Herbivores	100	1683<br>(144-4883)
Merlinius	Dolichodoridae	Herbivores	31.25	467<br>(114-921)
Neodolichorhynchus	Telotylenchidae	Herbivores	12.50	381<br>(320-441)
Paratylenchus	Tylenchulidae	Herbivores	62.5	566<br>(53-2375)
Pratylenchus	Pratylenchidae	Herbivores	18.75	67<br>(29-125)
Psilenchus	Tylenchidae	Herbivores	12.50	87<br>(48-127)
Trichodorus	Trichodoridae	Herbivores	25.00	106<br>(32-238)
Tylenchorhynchus	Telotylenchidae	Herbivores	31.25	2177<br>(64-9913)
Tylenchus	Tylenchidae	Herbivores	18.75	917<br>(141-1547)
Xiphinema	Longidoridae	Herbivores	18.75	73<br>(48-90)
Dorylaimus	Dorylaimidae	Omnivores	6.25	84<br>(84-84)
Aporcelaimellus	Aporcelaimidae	Predators	25.00	48<br>(4-127)
Aporcelaimus	Aporcelaimidae	Predators	56.25	174<br>(3-375)
Eudorylaimus	Qudsianematidae	Predators	6.25	21<br>(21-21)
Labronema	Dorylaimidae	Predators	6.25	3<br>(3-3)
Discolaimus	Discolaimidae	Predators	31.25	35<br>(1-59)
Mononchus	Mononchidae	Predators	6.25	41<br>(41-41)
Nygolaimus	Nygolaimidae	Predators	18.75	469<br>(240-874)

(a) Prevalence = percentage of samples in which a nematode genus was detected with respect to total number of samples processed.

(b) Soil nematode density = number of nematodes of each genus per 500 cm^3 of soil in all sampling points where that genus was detected, expressed as mean population (range, minimum and maximum).

Supplementary Table 2. Soil physicochemical properties of the six commercial almond groves. By row, different letters indicate significant differences according to Tukey test ($P < 0.05$). Mean and standard deviation was calculated for each locality (mean ± s.d.).

	Alcala del Rio	Alcolea	Carmona	Cordoba	Los Palacios	Marmolejo
**Ca (meq/100g)***	7.42 ± 0.982 a	3.77 ± 1.52 b	2.7 ± 0.677 b	11.3 ± 2.13 a	2.4 ± 0.566 b	7.53 ± 1.3 a
**CEC (meq/100g)**	11.4 ± 1.2 bc	7.37 ± 0.952 cd	5.62 ± 0.121 d	18.5 ± 3.95 a	5.24 ± 0.735 d	12.2 ± 1.1 b
**C:N**	9.76 ± 5.28	7.04 ± 2.31	8.07 ± 1.06	8.24 ± 0.771	8.24 ± 1.49	7.1 ± 3.63
**CO3 (%)**	23 ± 0.155 a	23.5 ± 4.51 a	0.373 ± 0.0924 b	24 ± 7.97 a	1.83 ± 0.332 b	24.5 ± 2.6 a
**EC (mS/cm)**	0.157 ± 0.00577 b	0.197 ± 0.0404 b	0.163 ± 0.0208 b	0.285 ± 0.0354 a	0.23 ± 0.0141 ab	0.223 ± 0.0321 ab
**K (meq/100g)**	0.46 ± 0.0173 bc	0.37 ± 0.0173 cd	0.207 ± 0.0404 d	1.14 ± 0.226 a	0.455 ± 0.0495 bcd	0.607 ± 0.0569 b
**Mg (meq100g)**	2.95 ± 0.4 ab	2.68 ± 0.9 ab	2.22 ± 0.548 ab	5.22 ± 1.52 a	1.72 ± 0.106 ab	3.48 ± 1.2 ab
**N (%)**	0.08 ± 0.01 ab	0.0633 ± 0.0231 bc	0.0367 ± 0.00577 c	0.105 ± 0.00707 a	0.055 ± 0.00707 bc	0.08 ± 0.01 ab
**Na (meq/100g)**	0.547 ± 0.101 ab	0.553 ± 0.0764 ab	0.493 ± 0.085 b	0.885 ± 0.0778 a	0.66 ± 0.0141 ab	0.46 ± 0.176 b
**OM (%)**	1.39 ± 0.797	0.783 ± 0.524	0.48 ± 0.0608	1.5 ± 0.099	0.795 ± 0.0495	1.01 ± 0.618
**P (mg/kg)**	4.07 ± 1.27	10.5 ± 4.09	21 ± 15.1	13.4 ± 8.84	18 ± 0.283	26.2 ± 14
**pH**	8.65 ± 0.103a	8.61 ± 0.139 a	5.67 ± 1.06 b	8.68 ± 0.113 a	8.86 ± 0.0212 a	8.41 ± 0.0755 a
**Clay (%)**	16.1 ± 1.44 bc	12.1 ± 1.45 bc	8.77 ± 1.34 bc	28.6 ± 9.90 a	5.5 ± 0.707 c	18.5 ± 2.43 ab
**Sand (%)**	35.7 ± 2.98 c	59.7 ± 5.68 b	81.6 ± 2.45 a	27.5 ± 15.4 c	89.6 ± 0.919 a	41.0 ± 5.50 c
**Silt (%)**	46.5 ±1.55 a	28.2 ± 4.23 b	9.67 ± 1.29 c	43.9 ± 5.52 a	4.95 ± 0.212 c	40.5 ± 6.56 a

*Abbreviations: Ca = Calcium, CEC = Cation Exchange Capacity, C:N = Carbon to Nitrogen ratio, CO3 = Carbonate, EC = electrical conductivity, K = Potassium, Mg = Magnesium, N = organic Nitrogen, Na = Sodium, OM = Organic Matter, P = available Phosphorous.
